# Density Matrix via Few Dominant Observables for the Ultrafast Non-Radiative Decay in Pyrazine

**DOI:** 10.1021/acs.jctc.2c01211

**Published:** 2023-01-19

**Authors:** Ksenia Komarova

**Affiliations:** The Fritz Haber Center for Molecular Dynamics and Institute of Chemistry, The Hebrew University of Jerusalem, Jerusalem91904, Israel

## Abstract

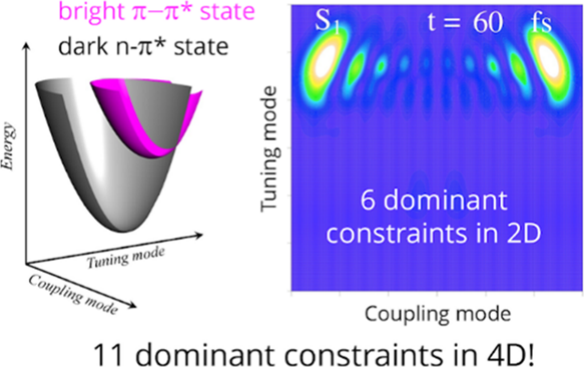

Unraveling the density matrix of a non-stationary quantum state as an explicit function of a few observables provides a complementary view of quantum dynamics. We have recently developed a practical way to identify the minimal set of the dominant observables that govern the quantal dynamics even in the case of strong non-adiabatic effects and large anharmonicity [Komarova et al., *J. Chem. Phys. 155,* 204110 (**2021**)]. Fast convergence in the number of the dominant contributions is achieved when instead of the density matrix we describe the time-evolution of the surprisal, the logarithm of the density operator. In the present work, we illustrate the efficiency of the proposed approach using an example of the early time dynamics in pyrazine in a Hilbert space accounting for up to four vibrational normal modes, {*Q*_10a_, *Q*_6a_, *Q*_1_, and *Q*_9a_}, and two coupled electronic states, the optically dark  and the bright  states. Dynamics in four-dimensional (4D) configurational space involve 19,600 vibronic eigenstates. Our results reveal that the rate of the ultrafast population decay as well as the shape of the nuclear wave packets in 2D, accounting only for {*Q*_10a_,*Q*_6a_} normal modes, are accurately captured with only six dominant time-independent observables in the surprisal. Extension of the dynamics to 3D and 4D vibrational subspace requires only five additional constraints. The time-evolution of a quantum state in 4D vibrational space on two electronic states is thus compacted to only 11 time-dependent coefficients of these observables.

## 1 Introduction

Correspondence between the classical observables and their respective quantal analogues, self-adjoint Hermitian operators, is one of the postulates of quantum mechanics. Dynamics of these observables can be computed in two equivalent ways. In the Schrodinger representation, it is given by the “state” function, the time-dependent wave function or, in a more general case, the time-dependent density matrix, ρ̂(*t*). The propagation of the initial state can be carried out using different techniques,^1−4^ and if we know the “state,” then we can compute the mean value of any observable: . In the Heisenberg picture, the time-evolution is carried by the observables and is explicitly governed by their commutation relations with the Hamiltonian. In a simple example of the dynamics on a harmonic potential,^5^ the time-evolution of the coordinate, *R̂*, and the momentum, *P̂*, operators are given by
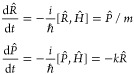
1

Here, *Ĥ* is the harmonic Hamiltonian, , and the square brackets denote the commutator, . Essentially, quantal non-commutativity between two operators, , results in the intertwining of their time-evolution. Equation 1 shows that no knowledge about the time-evolution of other observables is required to propagate ⟨*R̂*⟩ and ⟨*P̂*⟩—the time-evolution of  group of operators is closed under the commutation with the Hamiltonian, they form a closed Lie algebra. Existence of the dynamical symmetry between the observables uncovered within the algebraic treatment of the time-evolution is not unique to coordinate and momentum operators. There are few other closed Lie algebras important in the field of molecular physics, quantum electrodynamics, and extensively growing field of quantum computing.^6−10^

Complementary algebraic view on the dynamics opens up when the initial state in the Schrodinger representation is defined as an explicit function of the observables. Consider for example a thermal state, , where β = 1/*kT* is the inverse temperature,  is the Hamiltonian, and *Z*is the partition function. The unitary time-evolution of this density matrix is determined by the time-evolution of the observable :
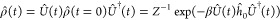
Here *Û*(*t*) denotes the unitary time-evolution operator.^11^ This manifests a unique algebraic route to the observables that govern the propagation of the initial state. It is this route that we follow in the present study to describe the internal conversion, one of the crucial processes in organic photochemistry.^12,13^

The thermal state is a special case of a more general type of distributions—states of maximal information entropy. We refer the reader to ref (14) for detailed historical overview regarding the concept of maximal entropy as there were many precursors before the main ideas were formed. The major boost was done in 1948, Claude Shannon in his work on the general theory of communication^15^ introduced the concept of information entropy as the measure of the information content. Several years after, in 1957 Edward Jaynes^16^ related Shannon’s formalism of information entropy to the entropy of a thermodynamic system by recognizing that the sole information about the microstate of a system consists of the values of the observables, macroscopic quantities. Following Shannon, Jaynes derived the least biased state as an exponential function of these observables, the state that maximizes the information content subject to the known mean values. Out of all possible probability distributions, the one that maximizes the entropy will have the largest amount of information, subject to the given knowledge about the system, subject to the given values of the observables as the constraints.

Fruitful integration of the information theory into statistical mechanics and thermodynamics^14,16−19^ inspired Raphael Levine and Richard Bernstein in the early 1970s to explore various aspects of the molecular collision phenomena from the perspective of information entropy.^11,20−23^ It was the first time the formalism of maximal entropy was used for molecular systems outside of equilibrium, chemical reactions. For a set of a three-center, atom-transfer exoergic reactions A + BC → AB* + C, the product energy distribution was analyzed in terms of its deviation from the most probable, prior, distribution.^20,21,24^ The choice was stimulated by the availability of the experimental results on the distribution of the product energy states by means of the molecular beam, chemiluminescence, and chemical laser techniques.^25−28^ In the series of studies,^20,21^ the experimental distribution of the final states was fitted with only one constraint per degree of freedom: translational, rotational, or vibrational.^21^ This ultracompact view was enabled by the specific definition of the constraint to be a fraction of the total energy in each degree of freedom relative to the overall accessible energy—a measure of the energy disposal. Later, the applicability of the formalism to the energy disposal accounting for the electronic degrees of freedom was also discussed for the same type of exoergic reactions.^23^

After a few years, an explicit algebraic view for the time-evolution of the state of maximal entropy was developed.^11^ The state of maximal entropy in general can be written as , where  are the observables and λ_*k*_ are respective Lagrange multipliers. The coefficients λ_*k*_ are termed the Lagrange multipliers in analogy to the problem of the maximization of the information entropy subject to the given mean values of the observables :
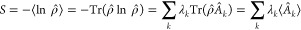
2

Basic constraint is that the state is normalized. In the example of a thermal state mentioned above, λ_0_ = ln *Z*, which is the Lagrange multiplier that assures the normalization. Here and in what follows, we define the density in a fully resolved set of quantum states, assuming no grouping of states. In such a case, in the absence of any other constraints beyond the normalization, the density matrix is an identity operator.

Under the unitary time-evolution, the state of maximal entropy remains exponential as a function of the constraints  with the essential difference that the constraints become time-dependent
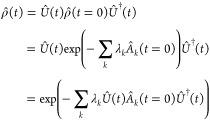
3

Since the density operator remains exponential throughout the time-evolution, the surprisal, the logarithm of the density matrix, , is a much simpler construct, in particular when constraints  do not commute. The initial surprisal is a simple linear function of the initial constraints: .

An important feature of the dynamics of the state of maximal entropy was uncovered^11^ for the specific case when the time-evolution of the constraints is closed under the commutation relation with the Hamiltonian
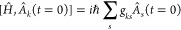
4here, *Ĥ* is the Hamiltonian that governs the time-evolution. In the case of closure, for every constraint , the sum on the right-hand side involves only the members of the finite set . The algebra of  operators discussed before, eq 1, is one of the simplest examples.

The closed algebra of the constraints allows us to analytically transform^11^ the set of the time-dependent constraints that govern the time-evolution,  in eq 3, into the set of time-independent constraints. Using eq 4, we can derive analytically the equations of motion for the Lagrange multipliers^11,29^
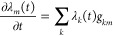
5

The dynamics of the surprisal, and thereby the density, is then governed only by the time-dependence of the Lagrange multipliers, λ_*k*_(*t*). Closed set of constraints is the key step toward representing the surprisal (and the density) as a linear combination of the time-independent observables: .

Recently, we extended^29^ this formalism of the dynamical theory developed for the propagation of the surprisal on one electronic state^11^ to the time-evolution of the surprisal that unfolds in the direct product Hilbert space of the electronic and the vibrational degrees of freedom. This approach allows us to compute the dynamics for systems that are essentially quantal, exploring various scenarios of the photochemical dynamics, for example when the coherent superposition of several electronic states is excited.^29,30^

In the proposed approach, the time-dependent surprisal *Î*(*t*) is computed in a matrix form using equations of motion for the initial surprisal defined in a finite basis. In this representation, the operators, {|*i*><*j*|}, for the basis functions *i* and *j*, form the least compact set that is closed under the commutation with the Hamiltonian. If the finite basis is large enough, this expansion is exact, and the algebra is closed. The matrix elements of the surprisal then play a role of the time-dependent Lagrange multipliers for the Gelfand states, {|*i*><*j*|}.

The possible expansion of the computed surprisal by a more compact set of time-independent observables requires help from the algebraic approach. We define a priori the time-independent observables  that are involved in the dynamics using algebraic equations of motion for the time-evolution of the initial set of constraints. The time-dependent coefficients of each of the observables, λ_*m*_(*t*), can then be calculated by taking the Hilbert–Schmidt product of each of the constraint  with the time-dependent surprisal computed in the same finite basis {|*i*⟩}_*i*=1,*N*_ of vibronic states
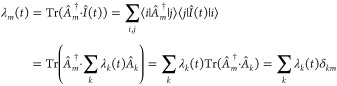
6here, we assume that the set of observables is orthonormal, see more details in ref (29). In a lot of cases of photochemical dynamics, the algebra of the initial set of constraints is not closed either due to electronic or vibrational coupling and/or anharmonic effects.^29,31^ The Lagrange multipliers in this situation can be found using eq 6 for an extended predefined set of operators, which will only approximate the exact surprisal. This approximate set is identified on the basis of algebraic equations of motion for the initial constraints. The number of the constraints that needs to be included in the approximate expansion often grows rapidly with the increase in the number and strength of the coupling parameters.

**Figure 1:**
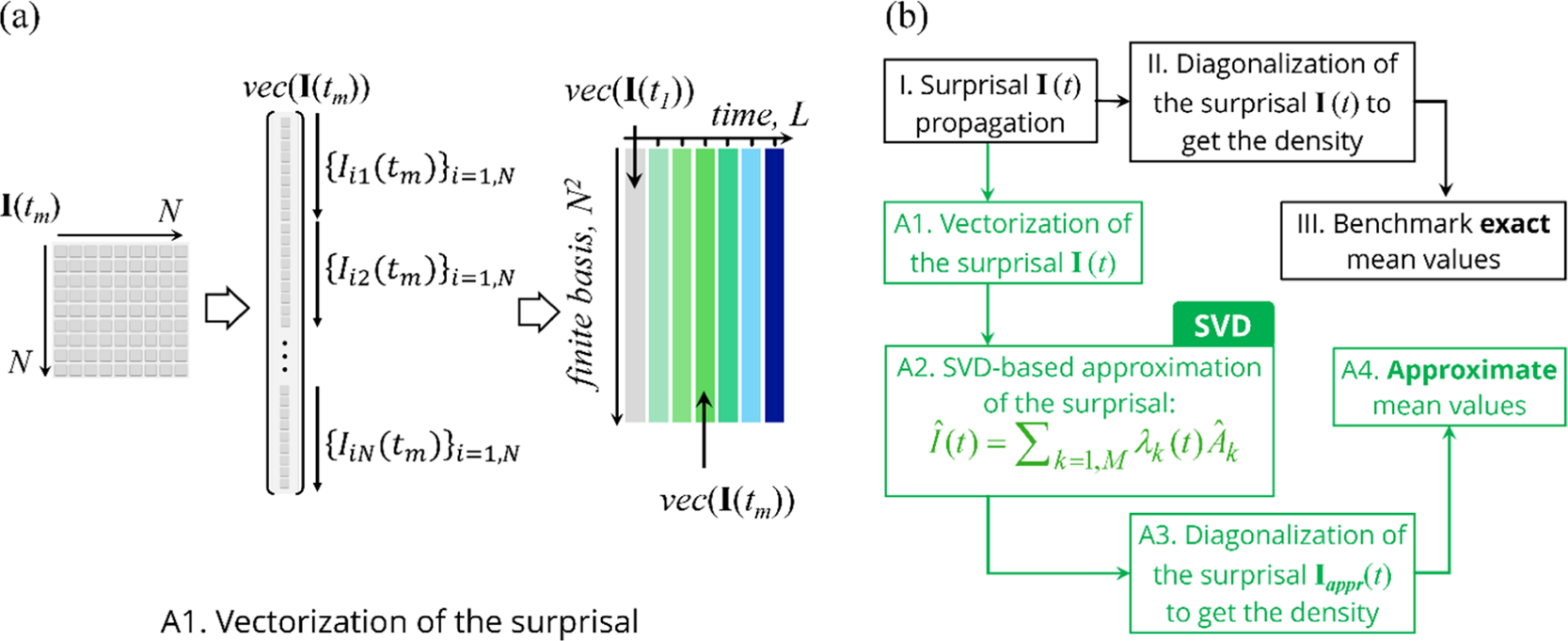
Computational steps toward approximate surprisal via *M* ≪ *L* dominant observables. (a) Vectorization of the surprisal matrices, **I**(*t*_*m*_), computed in *N* by *N* finite basis representation at *L* time points, *t*_*m*_. The dominant observables are evaluated via SVD of the resulting *N*^2^ by *L* rectangular matrix shown on the right. (b) Calculating the density and the respective mean values using exact (I–III, shown in black color) and approximate surprisal (A1–A4, shown in green color). The accuracy of the approximation is analyzed based on the comparison of the mean values for the electronic population, the nuclear coordinates, and their dispersion.

To address this challenge, we developed^31^ a more practical way to compute the dominant constraints. Inspired by the work on empirical surprisal analysis in complex biological systems,^32−34^ we apply the singular value decomposition (SVD) technique^35^ to the vectorized form of the time-dependent surprisal. Vectorization provides a vector of *N*^2^ matrix elements of the surprisal computed at time *t*_*n*_, see Figure 1. We stack the vectors defined for *L* time points sequentially as columns and form a rectangular matrix of the time-dependent surprisal, *N*^2^ by *L* matrix elements. This approach allows us to disentangle the time variable from the vibrational and electronic degrees of freedom, providing a fully numerical version for the algebraic expansion: **I**_appr_(*t*) = ∑_*k*=1,*M*_λ_*k*_(*t*)**Α**_*k*_. The number of the dominant constraints *M* determines the accuracy of the approximation. For a set of *L* time points at which the surprisal is computed, if *M* = *L*, the SVD expansion is essentially exact, however most often^29−31^*M* ≪ *L*.

The developed technique has a resemblance with the methods based on the propagation of the matrix product states, tree tensor networks, and density matrix renormalization group formalism useful in different quantum chemical/dynamical applications.^36−45^ These methods are formulated for the wave function of a quantum state, instead we consider here the time-evolution of the surprisal. The proposed route is highly efficient for the propagation of the states of maximal entropy (subject to constraints), providing exceedingly small number of the time-dependent coefficients for the coherent multi-electronic state dynamics.^29−31^

In the present work, we apply the SVD-based surprisal analysis^31^ to identify the set of dominant observables that drive the S_2_–S_1_ non-radiative transitions in pyrazine. Internal conversion in pyrazine is extensively studied by various theoretical^37,46−60^ and experimental methods.^61−68^ Similar to other heteroaromatic organic chromophores,^13^ strong non-adiabatic interaction between the bright S_2_(ππ*) and the dark S_1_(nπ*) electronic states results in the electronic relaxation during the ultrafast passage through the conical intersection region of the two potential energy surfaces (PES). There are evidences that more than two electronic states are involved in the overall relaxation process, see ref (68) and discussion therein. However, the two-states model^54^ is commonly accepted as a benchmark to compare the efficiency of various computational methods in the field of non-adiabatic quantum dynamics.^57,69,70^ We follow the two electronic states picture as our aim is to provide an alternative representation of the well-studied dynamics using an approximate description of the non-stationary in time density matrix by the time-independent observables.

The two-state model diabatic Hamiltonian is designed^46,47,51−54^ within the normal mode representation for all 24 vibrational degrees of freedom. Among them, there are four normal coordinates, , that are most strongly coupled while the rest modes acting as an intramolecular “bath.”^52,55^ The diabatic coupling between two electronic states is a linear function of the coupling coordinate *Q*_10a_—the out-of-plane motion of hydrogen atoms. Other modes, the so-called tuning modes, represent the nuclear motion that affects the S_2_–S_1_ energy gap between the PES toward the conical intersection.

Previous computational studies^48−50^ of the quantum dynamics in pyrazine and similar vibronic coupling systems show that the nuclear motion along the coupling and tuning modes is qualitatively different. While the nuclear wave packets along the tuning modes tend to be more localized in the vicinity of the respective mean values , the spreading of the nuclear wave packets as a function of the coupling mode coordinate is substantially higher. This makes it a good benchmark not only for the description of the ultrafast electronic population transfer but also for essentially quantal description of the nuclear motion.

Efficient numerical schemes^52,54,71^ of multi-dimensional time-dependent Hartree approach are available and enable description of the dynamics in pyrazine in the full 24-mode configurational space. We employ in the present work only the four-mode  diabatic Hamiltonian. Limiting the number of the nuclear degrees of freedom allows us to examine the accuracy of the surprisal approximation not only for electron dynamics but also for the description of the electron-nuclear motion along the coupling and tuning normal modes. The major aim is to understand what observables govern the time-evolution of the density in this typical example of strong vibronically coupled systems, and how these constraints change upon expansion of the nuclear configurational space.

We begin by computing the dynamics accounting for one coupling and one tuning mode,  (2D), and then extend the nuclear configuration space by adding other tuning modes into consideration,  (3D) and  (4D) sets of vibrational coordinates. Expansion of the nuclear Hilbert space when going from two to four nuclear degrees of freedom does not result in a dramatic growth in the number of the dominant constraints. Accurate representation for the S_2_ electronic population decay together with an extensive 4D nuclear dynamics is achieved via only 11 dominant observables.

The paper is organized as follows. Section 2 provides computational details of the SVD-based surprisal analysis. In Section 3, we discuss the accuracy of the dominant constraints approximation in 2D, 3D, and 4D dynamics in pyrazine. We analyze the rate of the electronic population exchange, Section 3.1, together with the description of the nuclear motion: mean values of the nuclear coordinates and their dispersion, Section 3.2. Comparison of the Lagrange multipliers and the dominant constraints computed for 2D, 3D, and 4D cases is given in Section 3.3. In Section 4, we summarize and discuss the concluding remarks.

## 2 Computational Details

We follow the approach developed and discussed in detail in ref (31), thereby we will only provide here the essential steps of the computational scheme, see Figure 1. In Section S1 of Supporting Information, we give additional technical details about the quantum dynamical calculations.

In order to identify the dominant observables that drive the non-radiative decay, we directly compute the time-dependent surprisal, the logarithm of the time-dependent density matrix . At the time *t* = 0, all the population is in the ground vibrational state of the bright S_2_(ππ*) diabatic state, with the mean values of the nuclear coordinates , see more details about the initial state in Section S1. The time propagation of the surprisal is determined^11^ using the Liouville–von Neumann equation of motion
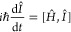
7here, *Ĥ*—is the diabatic Hamiltonian^54^ that governs the unitary time-evolution and includes the diabatic coupling between two PES, see Section S1 of Supporting Information. We compute the dynamics of the surprisal in the finite basis representation of *N* eigenstates of the diabatic Hamiltonian *Ĥ*. The eigenstate basis is generated using diagonalization of the Hamiltonian, initially defined in a zero-order basis of the non-shifted Hermite polynomials for each normal mode. For 2D dynamics, we use up to {20,30} for  harmonic basis functions, respectively, giving in total for two electronic states *N* = 20 × 30 × 2 = 1200 basis functions. In 3D case, we operate with {20,30,10} basis set for  modes, and finally, we perform 4-mode computation using up to {20,10,7,7} basis for  normal modes. Absorption spectra calculated for each model system in comparison to the experimental absorption^61^ are shown in Figure S1 in Supporting Information.

The major benefit of using the eigenstate basis is that the diagonal elements of the surprisal in this basis are constant, while the time-evolution of the off-diagonal elements is analytical
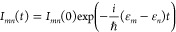
8where  are the eigenvalues of the Hamiltonian *Ĥ* and *I*_*mn*_(0) is an initial value of the surprisal matrix element.

Dominant observables and their coefficients are evaluated for a vectorized matrix of the time-dependent surprisal. The vectorization routine is implemented in a following way: for each time point of the dynamics, we reorganize the matrix elements of the surprisal, calculated using eq 8, into a vector by placing its columns one after the other, see Figure 1a. Then, we concatenate these column-vectors of the surprisal obtained for each time point sequentially, so that the columns follow  order. The resulting rectangular matrix has *N*^2^ rows and *L* columns, given the dynamics is computed for *L* time points.

This reorganization of the matrix of the surprisal allows us to disentangle the time and electron and nuclear variables via SVD procedure, step A2, Figure 1b
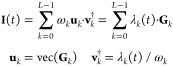
9Lagrange multiplier, λ_*k*_(*t*), is defined at each time point as the product of the respective singular eigenvalue ω_*k*_ and the component of the right eigenvector **v**_*k*_ at this time point. The right eigenvectors **v**_*k*_ are the eigenstates of the time-covariance matrix **T**
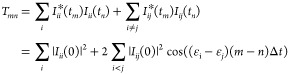
10here, we used the analytical expansion of the time-dependent surprisal matrix elements in the eigenstate basis of the Hamiltonian *Ĥ*, eq 8. The time-covariance matrix **T**, eq 10, is a real symmetric Toeplitz matrix—each descending diagonal of **T** from left to right is constant, thereby it is fully defined by its first row. Toeplitz matrices arise often^72−76^ in theory of weakly stationary processes, theory of signal processing, and information theory applications in statistical physics.

The left eigenvectors {**u**_*k*_} in eq 9 are the vectorized form of the constraints **G**_*k*_, which also can be written as *N* × *N* time-independent complex Hermitian matrices—observables. The number of terms in the expansion equals to the number of the time points, *L*, the lowest dimension of the rectangular matrix of the vectorized time-dependent surprisal. We select the dominant observables on the basis of the magnitude of singular eigenvalues ω_*k*_. Equation 9 is an exact expression, however as we will see, the expansion of the surprisal with a much fewer than *L* number of terms already captures the main features of the electronic and nuclear dynamics.

Experimental measurements^63,66^ suggest that the population of the bright state is almost fully transferred within 22 fs. Indeed, computational results for the four-mode dynamics show dramatic decay of the S_2_ population within 40 fs.^52,54,55^ We extend the computational time range up to 120 fs to ensure enough time for the system to explore the nuclear conformational space. For the case of 2D and 3D dynamics, we use a time step Δ*t* = 1 fs (*L* = 120), and in 4D dynamics, we increased the time step to Δ*t* = 5 fs (*L* = 24).

We benchmark the approximation of the surprisal via the finite set of constraints by comparing the exact and approximate mean values of the *i*th electronic state population, ⟨|*i*⟩⟨*i*|⟩, the mean values of the nuclear coordinates in each electronic state, , and their dispersion, , where *X* = 10a, 6a, 9a, 1. In the case of 2D dynamics, we also compare the shape of the nuclear wave packets: population distribution in each electronic state given as a function of nuclear coordinates  and .

Computation of the mean values and the population distribution requires evaluation of the density matrix for each time step of the dynamics, as shown in step III and A3 in the computational scheme in Figure 1b. Following the spectral decomposition of the time-dependent surprisal
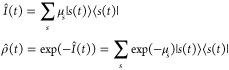
11the surprisal and the density share the same system of eigenvectors {|*s*(*t*)⟩}. Here, μ_*s*_ is the eigenvalue of the surprisal with the respective eigenvector |*s*(*t*)⟩. We diagonalize^77^ the matrix of the surprisal and use its eigenvalues and eigenvectors to reconstruct the density matrix. This numerical approach for surprisal to density transformation is valid also in the case when the observables do not commute.

Maps of the population distribution as a function of the normal mode coordinates on each electronic state are calculated by transformation of the eigenvectors {|*s*(*t*)⟩} to the zero-order basis of Hermite polynomials, , defined on a 2D grid of nuclear coordinates
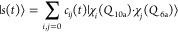
12

## 3 Results and Discussion

Internal conversion in pyrazine is an attractive benchmark model to test the performance of the SVD-based approximation of the surprisal given by the dominant observables. There are four nuclear degrees of freedom, one coupling and three tuning modes that are largely involved in the energy redistribution. The coupling mode induces rapid population transfer between the electronic states, while tuning modes govern an extensive nuclear motion in both excited PES. From the algebraic point of view, there is a multitude of operators that contribute to the time-evolution of the initial set of observables. Already in 2D case, in the first-order approximation, there are more than 20 operators, see discussion in Section S2 of Supporting Information. Hence, solving the dynamics of the Lagrange multipliers via the algebraic route becomes sophisticated. We illustrate the power of the numerical approach by comparing the SVD-based approximation of the surprisal in 2D, 3D, and 4D dynamics. As we will see, this expansion of the nuclear Hilbert space does not result in a dramatic increase in the number of the dominant contributions.

### 3.1 Electronic Population Transfer

**Figure 2:**
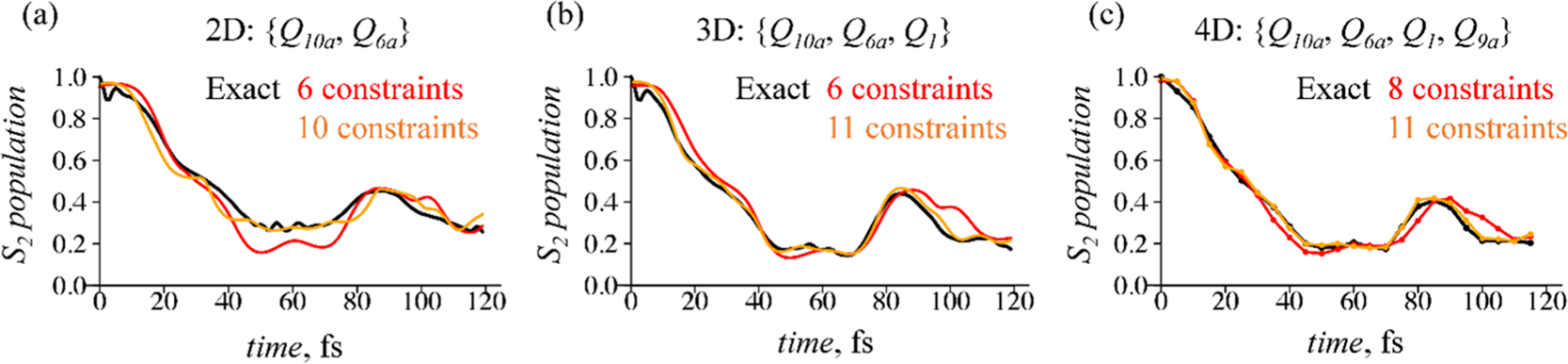
Population decay on the bright S_2_(ππ*) electronic state as a function of time computed for 2D (a), 3D (b), and 4D (c) nuclear dynamics. The vibrational coordinates involved in the dynamics are shown in curly brackets. The results are presented for two levels of the surprisal approximation via dominant constraints (red and orange lines, as shown in the legends) together with the benchmark exact population dynamics (black lines). Dynamics in 2D and 3D (a,b) are computed withΔ*t* = 1 fs, while in 4D, panel (c), the time step of Δ*t* = 5 fs was used.

Time-evolution of the population in S_2_(ππ*) electronic state is shown in Figure 2 separately for 2D, 3D, and 4D dynamics.

In the 2D  case, Figure 2a, 70% of the population is transferred already during the first 40 fs. Extension of the dynamics to the vibrational subspace with *Q*_1_ and *Q*_9a_ degrees of freedom even more facilitates the transfer, Figure 2b,c. In all three cases, there is also a weak back-transfer around 90 fs of the dynamics. We compare different levels of the surprisal approximation versus the exact results. Even the crude approximation of the surprisal given by only six time-independent observables (eight constraints in the case of 4D dynamics) well captures the rate of the electronic population exchange, Figure 2, and the back-transfer S_1_ → S_2_ around 90 fs. The higher level of approximation, orange line in all panels in Figure 2, provides even better agreement with the exact results throughout the time range of interest, showing fast convergence of the SVD-based approximation.

**Figure 3:**
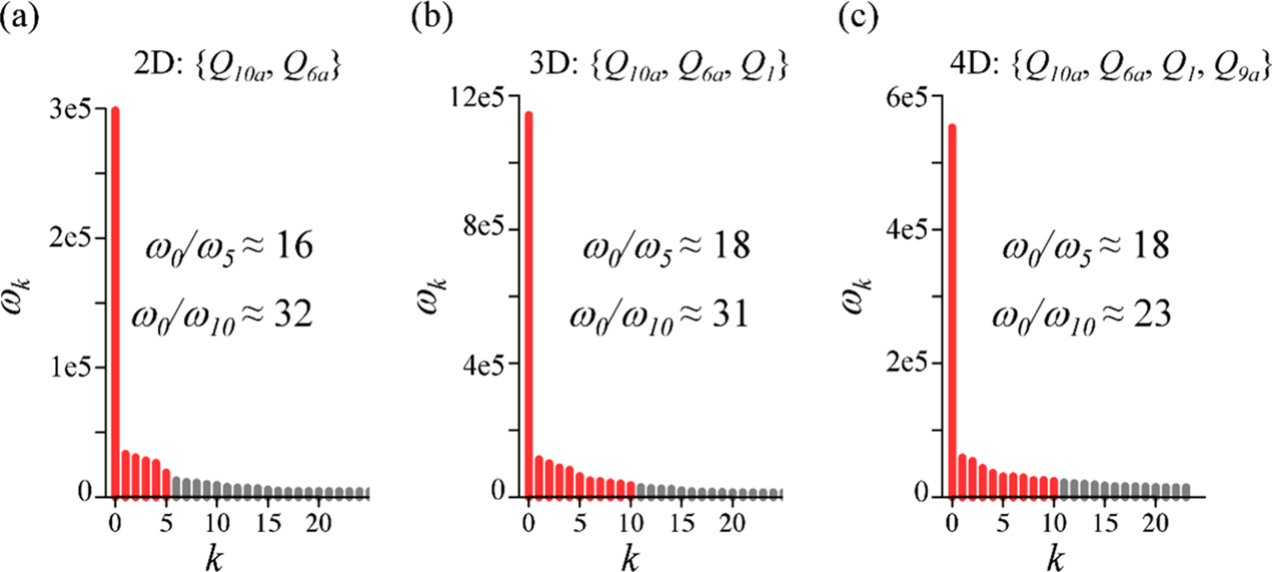
Singular eigenvalues in the SVD expansion of the time-dependent surprisal, eq 9, for the dynamics in 2D (a), 3D (b), and 4D (c) nuclear degrees of freedom. Normal mode coordinates involved in the dynamics are given on each panel. Red lines highlight the eigenvalues of the dominant constraints that we include in the approximation of the surprisal. In all three cases, the first eigenvalue ω_0_ is several orders of magnitude larger compare to other ω_*k*_, the relative ratio ω_0_/ω_*k*_ is shown on each panel.

We identify the number of terms in the SVD expansion of the surprisal, eq 9, by the magnitude of the singular eigenvalues ω_*k*_, as shown in Figure 3. See also Figure S2 in Supporting Information for the same data plotted on a logarithmic scale. One can see that in 2D case, there is a gap between the ω_5_ and higher eigenvalues, thereby we limit the number of terms in this case to six constraints. In 3D case, we compare two sets: a minimal one that involves six constraints and an extended set of 11 constraints. In 4D dynamics, the eigenvalues ω_5_ ≈ ω_6_ ≈ ω_7_ are almost degenerate, hence the minimal set consists of eight constraints and an extended set of 11 constraints.

### 3.2 Nuclear Motion: from 2D to 4D Dynamics

The rate of the electronic population decay and the time of the S_1_–S_2_ back-transfer are not changing significantly upon extension of the dynamics from a small Hilbert space of  normal modes to 3D and 4D dynamics. We can use the 2D case to examine the accuracy of the SVD-based approximation up to the fine details of the nuclear dynamics.

**Figure 4:**
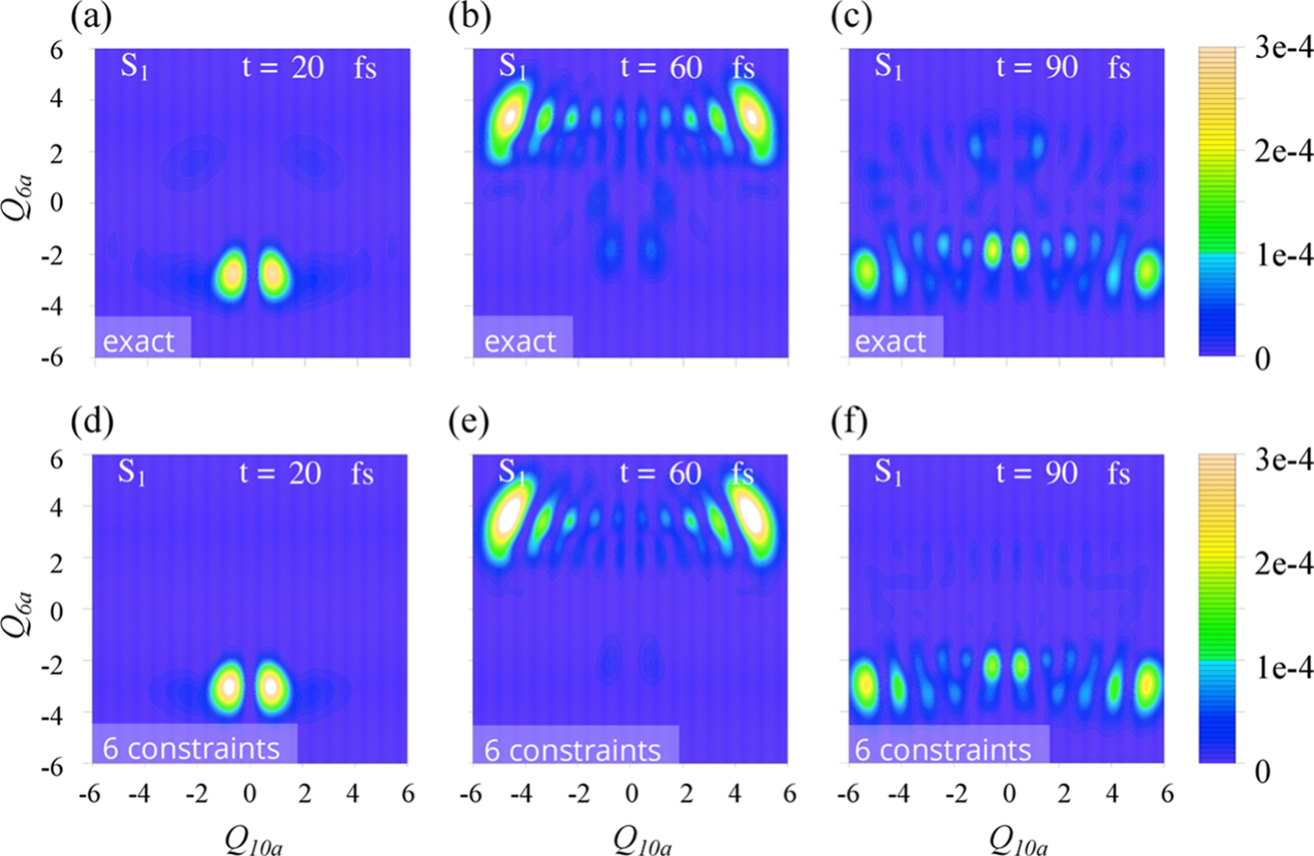
Snapshots of the population distribution in the 2D case of the dynamics on the dark, S_1_(nπ*), electronic state as a function of two nuclear coordinates: coupling mode, *Q*_10a_, and tuning mode, *Q*_6a_, taken at different times of the dynamics, as shown in the top right corner on each panel. Exact results (a–c) are compared with the approximate computation (d–f) via six dominant constraints.

In Figure 4, the maps of the population distribution in S_1_ for a given time point are shown as a function of  nuclear coordinates: exact dynamics, top panels, versus the minimal approximation via six dominant constraints, bottom panels. Similar comparison for the bright S_2_ electronic state is given in Figure S3 in Supporting Information. The dynamics of the population distribution along the 120 fs time range is also presented in Movies S1 and S2 in the Supporting Information. The results demonstrate remarkable level of accuracy of this most crude few terms approximation of the surprisal.

At the early times of the dynamics, the S_1_ nuclear wave packet keeps a rather simple Gaussian shape along the *Q*_6a_ coordinate, see Figure 4a,d and Movie S1 in Supporting Information. The dynamics on two PESs is strongly coupled; the wave packet on the dark state closely follows the path of the wave packet on the bright electronic state. The early time nuclear motion on both electronic states is directed toward the minimum on S_2_ PES in the region of negative *Q*_6a_ values.^54^

Dynamics along the coupling coordinate preserve the symmetry of the normal mode, thereby the mean value  is constant throughout the time range studied. Spread of the nuclear wave packet along this coordinate is increasing rapidly between 20 and 60 fs exhibiting also a clear nodal structure, Figure 4b. It is the timeframe when the mean value ⟨*Q*_6a_⟩ of the 2D nuclear wave packet approaches the region of crossing^47^ of the two diabatic PESs. Approximate computation reproduces this essentially quantal feature in the nuclear motion, as compared vertically in Figure 4b,e panels.

In order to understand the origin of the nodal structure seen in the shape of the nuclear wave packets, we analyze the time-dependent vibrational state distribution in both electronic states, see Figure S4 and Movie S3 in Supporting Information. At the early times, vibrational levels of the coupling mode are only weakly excited in both electronic states. Approaching the region of the small energy gap between two diabatic PES at 20–30 fs leads to an increase in the population transfer toward highly excited vibrational states of the coupling mode. In the diabatic picture, the effective coupling is stronger for a region with a smaller energy gap,^78^ thereby we see more efficient transfer. The transfer of the population back to the bright S_2_ electronic state at 80–90 fs, Figure 2, occurs when the nuclear wave packets revisit this region of strong effective coupling.

**Figure 5:**
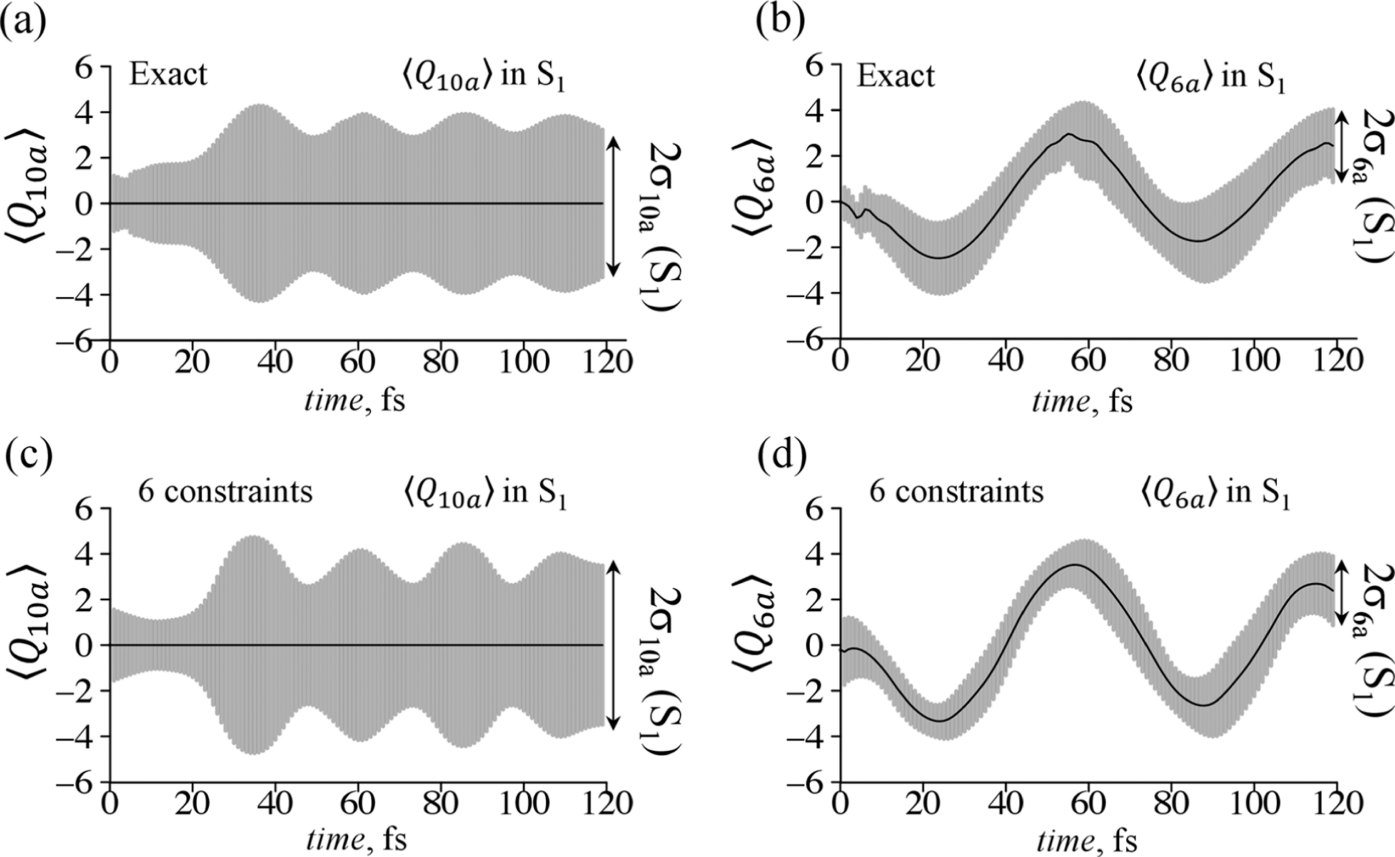
Dynamics of the mean values ⟨*Q*_10a_⟩ and ⟨*Q*_6a_⟩ (solid lines) and their respective dispersion σ_10a_ and σ_6a_ (gray area covering ) computed in the S_1_ electronic state for the 2D case. Exact computations (a,b) are compared to the approximation (c,d) given by six dominant constraints. The respective results computed for S_2_ electronic state are shown in Figure S5 in Supporting Information.

This feature of the vibronic energy redistribution has specific signatures in the time-evolution of the dispersion of the coupling coordinate, , see Figure 5a,c for its dynamics in S_1_ electronic state.

In contrast to almost classical picture for the dispersion of the tuning mode coordinate, Figure 5b,d, the coupling mode has rapid increase in σ_10a_, Figure 5a, at the time range when the nodal structure in the nuclear wave packets is observed. This rapid change in the dispersion is well captured in the approximate computation by six dominant constraints, Figure 5c. Similar features are also seen in the time-evolution of the dispersion in the second electronic state, as shown in Figure S5 in Supporting Information.

**Figure 6:**
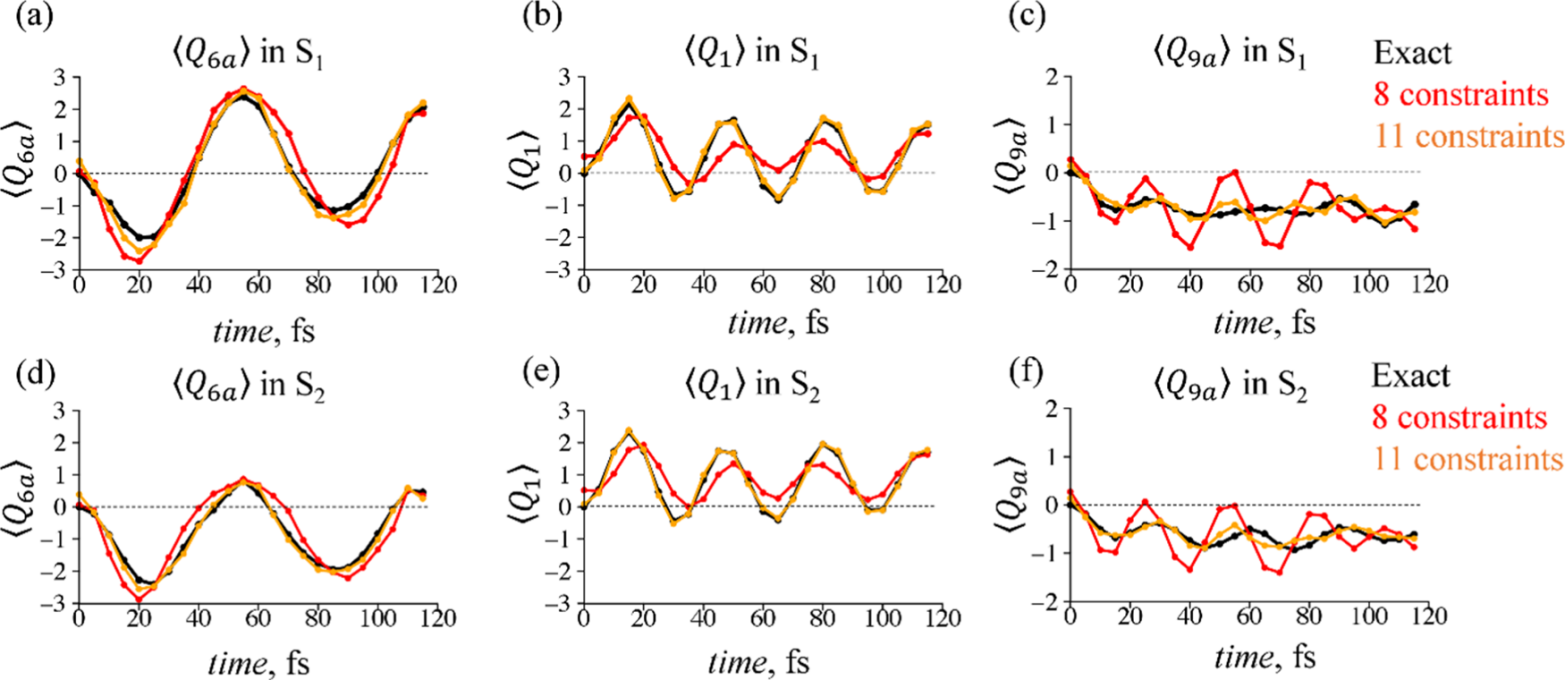
Time-dependent mean values of the tuning modes: ⟨*Q*_6a_⟩ (a,d), ⟨*Q*_1_⟩ (b,e), and ⟨*Q*_9a_⟩ (c,f) computed in S_1_ (a–c) and S_2_ (d–f) electronic states for the case of 4D dynamics. The results are presented for two levels of the surprisal approximation via dominant constraints: eight constraints (red line) and 11 constraints (orange line). The respective mean values from the benchmark exact computation are shown as black lines.

We analyze the accuracy of the approximation for 3D and 4D nuclear motion by computing the mean values of the nuclear coordinates and their dispersion. Comparison between the approximate and exact dynamics for the mean values of the three tuning modes in 4D vibrational Hilbert space is shown in Figure 6. The results for the 3D dynamics are given in Figures S6–S7 in Supporting Information.

**Figure 7:**
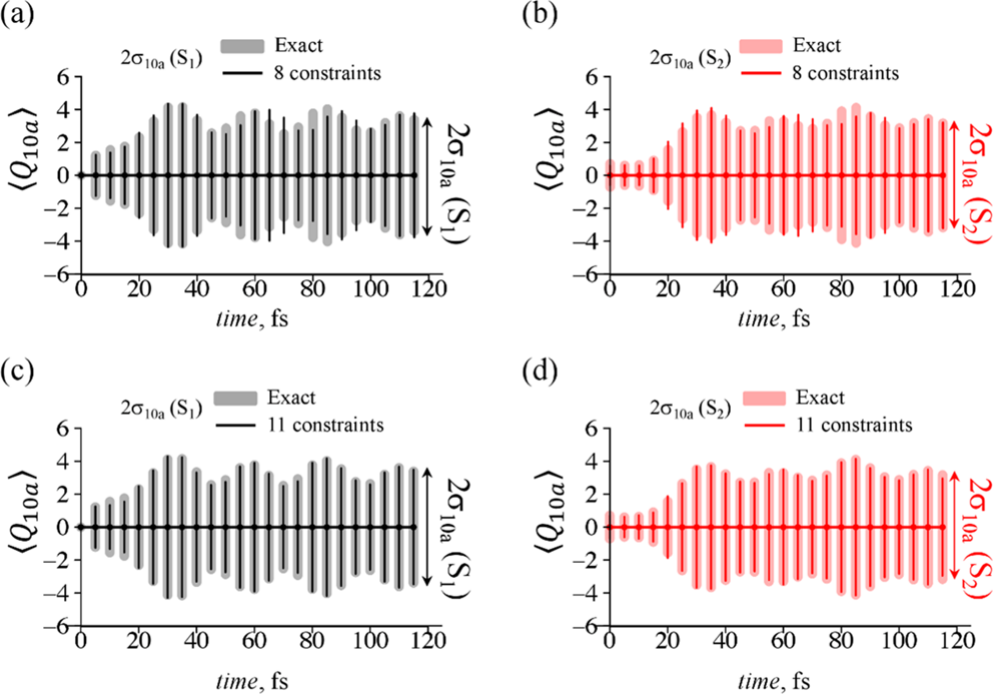
Time-evolution of the dispersion of the coupling normal mode, σ_10a_, in S_1_ (a,c) and S_2_ (b,d) electronic states for the case of 4D dynamics. Approximate results provided by eight (solid lines, a,b) and 11 (solid lines, c,d) dominant constraints are compared to the results of the exact computation (gray/red area covering ).

For the case of 4D dynamics, the minimal set of eight dominant constraints in the approximation of the surprisal qualitatively captures the nuclear motion along the tuning mode coordinate *Q*_6a_, Figure 6a,d. The dispersion in the coupling mode coordinate σ_10a_, Figure 7a,b, is also well reproduced. However, this approximation is clearly not sufficient to describe ⟨*Q*_1_⟩ and ⟨*Q*_9a_⟩ dynamics, Figures 6b,c,e,f and S8–S10 in Supporting Information.

Despite the not fully accurate representation of the nuclear motion, we see rather good level of description for the electronic population transfer, Figure 2c. This suggests higher importance of the nuclear dynamics along *Q*_6a_ and *Q*_10a_ coordinates in the process of the non-radiative decay. Similar trends we see in 3D dynamics, where the minimal set consists of only six dominant constraints (Figures S6–S7 in Supporting Information).

An extended, 11 constraints, expansion of the surprisal for 3D and 4D dynamics already shows much better description of the ⟨*Q*_1_⟩, ⟨*Q*_9a_⟩ mean values and their dispersion, see Figures 6, 7 and Figures S6–S10 in Supporting Information. Such a rapid convergence in the number of the dominant constraints was also seen for the dynamics on a single 1D Morse potential,^31^ where the algebra is not closed due to anharmonicity. At most, 14 constraints were required to provide a high level of the SVD-based approximation for the population dynamics among 30 vibrational eigenstates. In the present work, we obtain a much higher degree of compaction. In the case of 4D dynamics, the propagation of the initial state involves *N* = 19,600 vibronic basis functions, eigenstates of the diabatic Hamiltonian defined for two electronic states. Instead, the time-evolution is compacted to only 11 time-dependent coefficients of the time-independent dominant observables. This unprecedent degree of compaction is close to analytical description of the dynamics, when only very few terms are varying throughout the time-evolution. Next section provides a discussion of these components and their comparison for 2D, 3D, and 4D cases.

### 3.3 Lagrange Multipliers and the Dominant Constraints

Lagrange multipliers  are defined as the product of the respective singular eigenvalue ω_*k*_ and the components of the eigenvector **v**_*k*_ of the time-covariance matrix **T**, eqs 9 and 10. The magnitude of the Lagrange multipliers is governed essentially by the magnitude of the singular eigenvalues {ω_*k*_}, Figure 3, since the eigenvectors **v**_*k*_ are normalized. It is the time-dependence of λ_*k*_(*t*) that is fully controlled by the eigenvector **v**_*k*_ of the time-covariance **T** matrix.

The leading term in the SVD expansion of the surprisal is the dominant observable **G**_0_ with the largest singular eigenvalue, ω_0_ (Figure 3). Lagrange multiplier λ_0_ for this constraint is constant in time up to 1% of its value for all three cases of 2D, 3D, and 4D dynamics, see Figure S11 in Supporting Information. In the basis of the eigenstates of the molecular Hamiltonian *Ĥ*, this constraint is the only term that largely contributes to the diagonal values of the surprisal matrix. Comparison of the diagonal entries of **G**_0_ with the eigenvalues of the Hamiltonian *Ĥ* is given in Figure S12 in Supporting Information separately for 2D, 3D, and 4D dynamics. In the case if all the rest Lagrange multipliers were zero, the approximation **I** ≈ λ_0_**G**_0_ represents a state stationary in time. The surprisal and the density matrix in such a case are diagonal in the eigenstate basis of *Ĥ*. Hence, the contribution of other constraints to the dynamics can be regarded as the deviation of the dynamics from the steady state.

**Figure 8:**
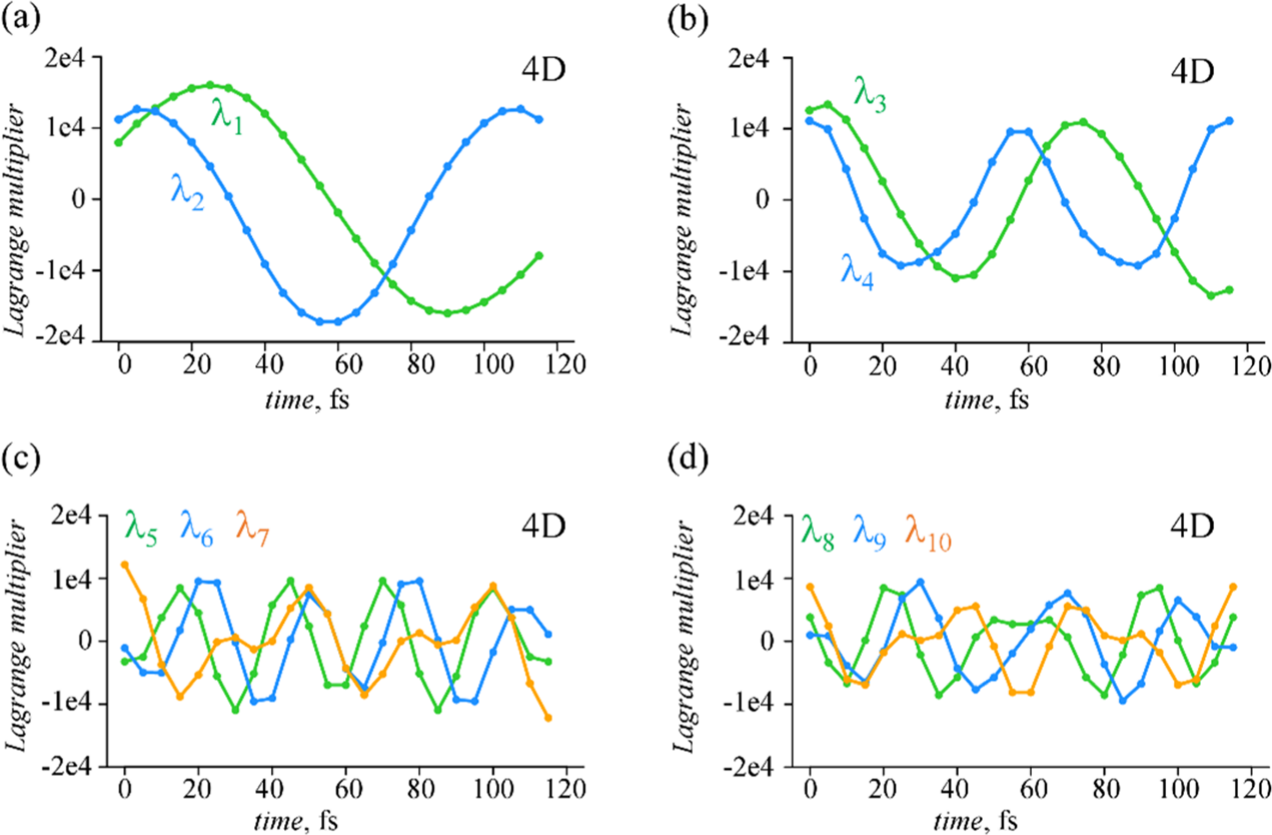
Time-dependent Lagrange multipliers, λ_*k*_(*t*), in the case of 4D nuclear dynamics in two electronic states for an extended set of 11 dominant constraints: (a) λ_1_(*t*) and λ_2_(*t*); (b) λ_3_(*t*) and λ_4_(*t*); (c) λ_5_(*t*), λ_6_(*t*) and λ_7_(*t*); and (d) λ_8_(*t*), λ_9_(*t*) and λ_10_(*t*). The Lagrange multiplier for the first constraint **G**_0_ is constant in time, see Figure S11 in Supporting Information. 4D vibrational space involves {*Q*_10a_, *Q*_6a_, *Q*_1_, and *Q*_9a_} normal modes.

Time-dependent Lagrange multipliers for the set of 11 dominant constraints computed for the case of 4D dynamics are shown in Figure 8, and λ_*k*_(*t*) for 2D and 3D cases are given in Figures S13–S14 in Supporting Information. In contrast to the coefficient of the leading term, λ_0_, the Lagrange multipliers with *k* > 0 oscillate in time around zero with increasing frequencies as the index *k* increases. The time-profile of the Lagrange multipliers with *k* = 1, 4 is similar while comparing dynamics with different numbers of nuclear degrees of freedom, Figures 8 and S13–S14 in Supporting Information.

**Figure 9:**
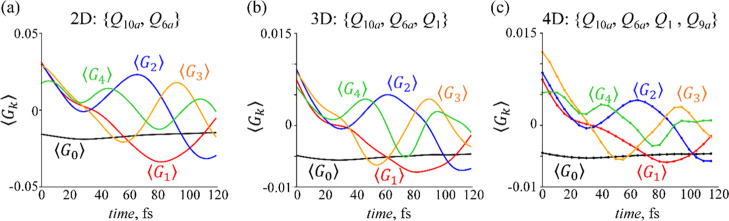
Time-evolution of the mean values of the first five dominant observables computed for 2D (a), 3D (b), and 4D (c) dynamics: ⟨*G*_0_⟩—black line, ⟨*G*_1_⟩—red line, ⟨*G*_2_⟩—blue line, ⟨*G*_3_⟩—yellow line, and ⟨*G*_4_⟩—green line. The magnitude of the mean values of the constraints is varying for 2D, 3D, and 4D cases; however, their time-profile is similar, as compared to the lines of the same color.

Time-dependent mean values of **G**_0_ to **G**_4_ dominant observables are shown in Figure 9. The mean value ⟨*G*_0_⟩ only weakly depends on time; as **G**_0_ is mostly diagonal on the basis of the Hamiltonian eigenstates, it is a constant of motion. For the constraints **G**_1_ to **G**_4_, the mean values are changing in time more rapidly. The time-profiles of the ⟨*G*_1_⟩ to ⟨*G*_4_⟩ mean values for 2D, 3D, and 4D dynamics have a similar pattern, as compared to the lines of the same color in Figure 9. This suggests that qualitatively, the constraints with the highest singular eigenvalues do not change much upon extension of the vibrational Hilbert space.

**Figure 10:**
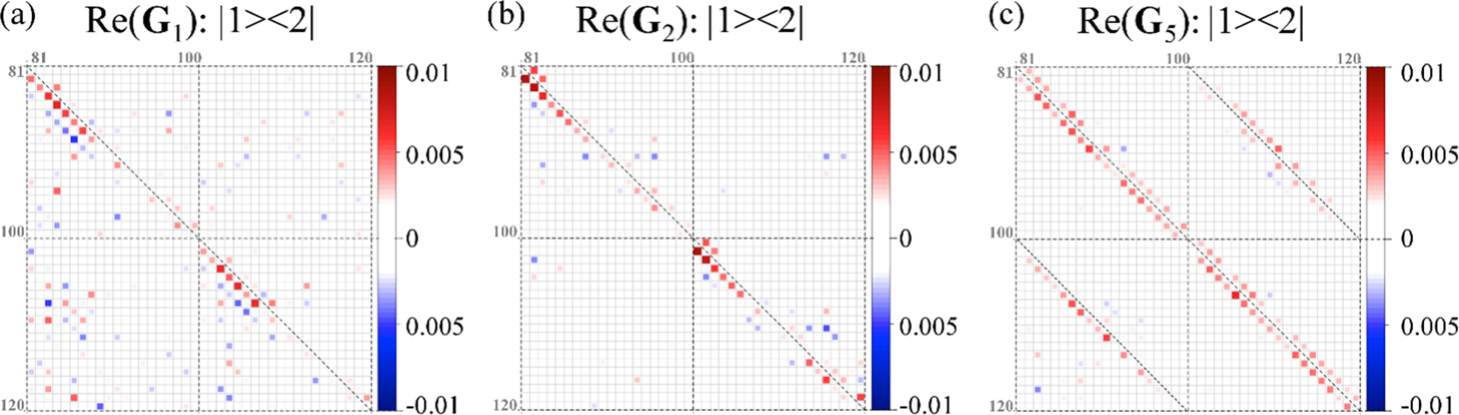
Vibronic coherence in the dominant constraints computed for 2D dynamics. The real part of the matrix elements in |1⟩⟨2| blocks off-diagonal in the electronic index are shown for **G**_1_ (a), **G**_2_ (b), and **G**_5_ (c) constraints. Corresponding indices of the matrix elements, 20·ν_6a_ + ν_10a_ + 1, are shown in gray on the sides of each block. Matrix elements with 81–100 indices correspond to the vibrational basis function with ν_6a_ = 4 of the tuning mode and ν_10a_ = 0,...,19 of the coupling mode. The elements with indices 101–120 are related to ν_6a_ = 5 and ν_10a_ = 0,...,19. The matrix elements of the dominant constraints spanning the full range of the vibrational states involved in the dynamics are given in Supporting Information, see Figures S15–S19.

For the case of 2D dynamics, we can examine the structure of the dominant constraints in more details. In the basis of vibronic states, the constraints have matrix elements that carry three indices: an electronic state index *i* = 1, 2; a vibrational index, ν_10a_ = 0,...,19, for the coupling mode *Q*_10a_; and an index, ν_6a_ = 0,...,29, for the tuning mode *Q*_6a_. To present the results, we arrange the matrices in blocks, so that the elements of these matrices are labeled as: 20·ν_6a_ + ν_10a_ + 1 for each |*i*⟩⟨*j*| block in the electronic index (*i*, *j* = 1, 2). Figure 10 shows an excerpt of four blocks of the matrix elements of the constraints **G**_1_, **G**_2_and **G**_5_ that contribute to the surprisal matrix elements off-diagonal in the electronic index, |1⟩⟨2| blocks. The indices of the blocks shown go from 81 to 120, ν_10a_ = 0,...,19 and = 4, 5; these vibronic states are strongly involved in the dynamics on both electronic states, see Movie S3 in Supporting Information. Matrix elements of the dominant constraints **G**_1_ to **G**_5_ computed in 2D case are given in Figures S15–S19 for all vibrational states involved in the dynamics.

Dominant constraints identified by the SVD approach are sparse and have non-zero matrix elements only for specific groups of states. For example, the matrix elements of the coherence in **G**_1_, **G**_2_ observables, Figure 10a,b, can be attributed to the combination of the diabatic coupling terms,  operators, as their non-zero matrix elements are confined to the first sub- and super-diagonal. However, only vibrational basis functions of the coupling mode with ν_6a_ = 0–8 contribute strongly. Constraint **G**_5_, Figure 10c, has symmetry analogous to the quadratic term where the diabatic coupling is multiplied by the shift operator in the tuning mode index,  kind of terms. The values of the **G**_5_ matrix elements also do not change monotonically with the increase in the vibrational index of the coupling mode. This complex structure of the dominant constraints, largely based on the spectral decomposition of the time-covariance matrix **T** of the surprisal, allows more efficient compaction of the dynamics providing much fewer terms compare to the strict algebraic derivation, see Section S2 in Supporting Information. Extension of the time-span of the dynamics up to 1 ps results in higher resolution in the time-covariances of the surprisal, giving very localized structure of the constraints, see Section S4 in Supporting Information.

## 4 Concluding Remarks

Expansion of the time-evolving state of maximal entropy (subject to initial constraints) as a function of time-independent constraints but with time-dependent weights, , enables a complementary compact view of quantal dynamics. The benefit is seen in particular at the early times of the quantal propagation when the set of dominant observables  remains small even for the more challenging case of an open algebra. This significantly facilitates the analysis of the quantal time-evolution: the effects of various parameters of the time-dependent Hamiltonian (such as parameters of the laser field, mass of nuclei, etc.) on the subsequent dynamics can be traced easily by comparing the changes in the time-dependence of the Lagrange multipliers, λ_*k*_(*t*), and the constraints, .

We identify the dominant constraint representation of the quantum dynamics that unfolds on two electronic states of pyrazine. We compare the Lagrange multipliers and the constraints for the dynamics in {*Q*_10a_, *Q*_6a_}, {*Q*_10a_, *Q*_6a_, *Q*_1_}, and {*Q*_10a_, *Q*_6a_, *Q*_1_, *Q*_9a_} nuclear Hilbert spaces. Electronic population decay and the nuclear motion along *Q*_10a_ and *Q*_6a_ normal coordinates have a very similar pattern when comparing 2D, 3D, and 4D case. We see this similarity reflected in the dominant constraint representation. The Lagrange multipliers of the first five constraints have similar time-profiles in 2D, 3D, and 4D cases. Also, the time-dependence of the mean values of these constraints is similar, see Figure 10. This suggests that with the major contribution to the surprisal, the dominant constraints **G**_0_ to **G**_4_ are not affected significantly upon addition of *Q*_1_ and *Q*_9a_ tuning modes.

The physical notion of the constraints and respective Lagrange multipliers still needs to be examined in order to design a dominant constraint representation for larger systems, where exact solution for the time-dependent surprisal is not possible. We already see that as in the case of the open algebra in anharmonic dynamics,^31^ the largest Lagrange multiplier belongs to the constraint that is diagonal on the basis of Hamiltonian eigenstates, the time-independent steady-state contribution to the exact surprisal. The constraints with *k* > 0 are sparse matrices and have only off-diagonal matrix elements on the basis of Hamiltonian eigenstates, and hence, they describe the deviation of the time-dependent surprisal from the time-independent steady state. Similar to the anharmonic case, we observe that only specific localized groups of basis states contribute to a particular constraint. The time-correlation in the contribution to the time-dependent surprisal for each group of states is tightly related to the eigensystem of the time-covariance matrix **T**, eq 10, and gives fruitful ground for future research.

## Supporting Information Available

The dynamics of the population distribution on S_1_ electronic state as a function of two nuclear coordinates along the 120 fs time-range for 2D case; left panel shows results of the 6 constraint SVD based approximation, while the right panel presents exact dynamics; normal mode coordinates are: q1 – coupling mode *Q*_10a_, q2 – tuning mode *Q*_6a_ (Movie S1) (MP4)
The dynamics of the population distribution on S_2_ electronic state as a function of two nuclear coordinates along the 120 fs time-range for 2D case; left panel shows results of the 6 constraint SVD based approximation, while the right panel presents exact dynamics; normal mode coordinates are: q1 – coupling mode *Q*_10a_, q2 – tuning mode *Q*_6a_ (Movie S2) (MP4)
Population dynamics on both electronic states for different vibrational basis functions in the 2D case: S_1_ population - black sticks, S_2_ population - red sticks; the index of the vibrational states is defined as 20·ν_6a_ + ν_10a_ + 1 , where ν_6a_ is the vibrational quantum number of the tuning mode, and ν_10a_ is the quantum number of the coupling mode; plotted is the population for the ν_6a_ = 0,...,14 and ν_10a_ = 0,...,19 (Movie S3) (MP4)
Technical details about quantum dynamical simulations; parameters of the diabatic Hamiltonian and the initial surprisal; detailed view on the algebraic equations of motion for the initial constraints; additional figures and short description for the singular eigenvalues and the constraints in the long-time-span limit (PDF)
