# Supplementary material for: Density Matrix via Few Dominant Observables for the Ultrafast Non-Radiative Decay in Pyrazine
Source: J Chem Theory Comput. 2023 Jan 19;19(3):746–57. doi: 10.1021/acs.jctc.2c01211 (PMC11137821; doi:10.1021/acs.jctc.2c01211)
Supplement: Supplementary file 4 — ct2c01211_si_004.pdf [file ct2c01211_si_004.pdf]

SUPPORTING INFORMATION

Density matrix via few dominant observables for the ultrafast non-radiative decay in pyrazine

Ksenia Komarova

The Fritz Haber Center for Molecular Dynamics and Institute of Chemistry,
The Hebrew University of Jerusalem, Jerusalem 91904, Israel

Contents:

Section S1. Computational details 2

Section S2. Algebraic equations of motion for the surprisal 4

Section S3. Additional figures 7

Section S4. Dominant constraints identified for the long time-span of the dynamics 22

Section S1. Computational details

Hamiltonian. We use the model diabatic Hamiltonian developed in Ref. 1 with only four vibrational normal modes taken into account: one coupling mode of B_{1g} symmetry, ν_{10a}, and three A_g tuning modes: $\nu_{6a}, \nu_1, \nu_{9a}$. The Hamiltonian is explicitly given by:

$$\begin{aligned}\hat{H} = &\sum_X \omega_X (\hat{a}_X^\dagger \hat{a}_X + \frac{1}{2}) \cdot \hat{\mathrm{I}} + \begin{pmatrix} -\Delta & 0 \\ 0 & \Delta \end{pmatrix} \\ &+ \sum_{X \in G_1} \frac{1}{\sqrt{2}} (\hat{a}_X + \hat{a}_X^\dagger) \begin{pmatrix} \alpha_X & 0 \\ 0 & \beta_X \end{pmatrix} + \sum_{X,Y \in G_1} \frac{1}{2} (\hat{a}_X + \hat{a}_X^\dagger)(\hat{a}_Y + \hat{a}_Y^\dagger) \begin{pmatrix} \alpha_{X,Y} & 0 \\ 0 & \beta_{X,Y} \end{pmatrix} \\ &+ \frac{1}{\sqrt{2}} (\hat{a}_{10a} + \hat{a}_{10a}^\dagger) \begin{pmatrix} 0 & \gamma_{10a} \\ \gamma_{10a} & 0 \end{pmatrix} + \sum_{Y \in G_1} \frac{1}{2} (\hat{a}_{10a} + \hat{a}_{10a}^\dagger)(\hat{a}_Y + \hat{a}_Y^\dagger) \begin{pmatrix} 0 & \gamma_{10a,Y} \\ \gamma_{10a,Y} & 0 \end{pmatrix}\end{aligned} \quad \text{(S1)}$$

We apply atomic units throughout. Here $\hat{a}_X^\dagger / \hat{a}_X$ are the creation/annihilation operators for the harmonic mode *X*. The G_1 group of modes involves only the tuning modes, $G_1 = \{\nu_{6a}, \nu_1, \nu_{9a}\}$. Equilibrium position of the harmonic potentials for the tuning modes is shifted from zero. This gives rise to the linear coupling terms diagonal in the electronic index. Diabatic coupling is induced only due to the nuclear motion along the coupling mode, ν_{10a}. In addition, there are quadratic coupling terms both diagonal and off-diagonal in the electronic index. The numerical values of the linear and quadratic coupling parameters, $\{\alpha_X, \beta_X, \gamma_X, \alpha_{XY}, \beta_{XY}, \gamma_{XY}\}$, are given in Tables S1-S2. The gap between the two electronic states, $\Delta = 0.423$ eV, and $\hat{\mathrm{I}}$ denotes the identity operator in the electronic subspace.

Table S1. Frequencies and linear coupling parameters (in eV) used in Eq. (S1).

X	*6a*	*1*	*9a*	*10a*
ω_X	0.0739	0.1258	0.1525	0.1139
α_X	-0.0981	-0.0503	0.1452	-
β_X	0.1355	-0.171	0.0375	-
γ_X	-	-	-	0.208

Table S2. Quadratic coupling parameters (in eV) used in Eq. (S1).

Y / X	*6a*	*1*	*9a*	*10a*
$\alpha_{X,Y}$				
6a	-	0.000984	-0.001863	-
1	0.000984	-	0.004309	-
9a	-0.001863	0.004309	-	-
10a	-	-	-	-0.01028
$\beta_{X,Y}$				
6a	-	-0.002714	-0.001726	-
1	-0.002714	-	0.001409	-
9a	-0.001726	0.001409	-	-
10a	-	-	-	-0.01028
$\gamma_{X,Y}$				
10a	-0.01	-0.004940	0.001142	-

Initial state. We consider an initial state that is fully localized on the bright S_2 electronic state and is centered at $Q_{10a} = Q_{6a} = Q_1 = Q_{9a} = 0$, the ground vibrational state of the non-shifted oscillators. The initial surprisal is diagonal in the zero-order basis of the non-shifted uncoupled oscillators:

$$\hat{I}(0) = -\ln \hat{\rho}_0 = \lambda_N + \lambda_0^{11} |1\rangle\langle 1| + \lambda_0^{22} |2\rangle\langle 2| + \sum_X \left(\lambda_X^{11} |1\rangle \hat{a}_X^\dagger \hat{a}_X \langle 1| + \lambda_X^{22} |2\rangle \hat{a}_X^\dagger \hat{a}_X \langle 2| \right) \quad \text{(S2)}$$

Here $X = \{10a, 6a, 1, 9a\}$. For 2D and 3D cases $X = \{10a, 6a\}$ and $X = \{10a, 6a, 1\}$, respectively. The values of the Lagrange multipliers are given by:

$$\begin{aligned} \lambda_N &= \ln Z + \frac{1}{2} \sum_X \beta \omega_X \\ \lambda_0^{11} &= -\lambda_0^{22} = \beta \Delta \\ \lambda_X^{11} &= \lambda_X^{22} = \beta \omega_X \\ Z &= \frac{\cosh(\beta \Delta)}{\prod_X 2 \sinh\left(\frac{1}{2} \beta \omega_X \right)} \end{aligned} \quad \text{(S3)}$$

The partition function, Z, is determined by the constraint of normalization. Parameter β is set to 8000 a.u.$^{-1}$ in order to approach a pure state initial distribution in all normal modes:

$$\begin{aligned}\rho_0 &= \exp(-\hat{I}(0)) \\ &= \exp\left[-\lambda_N - \lambda_0^{11}|1\rangle\langle 1| - \lambda_0^{22}|2\rangle\langle 2| - \sum_X \left(\lambda_X^{11}|1\rangle \hat{a}_X^\dagger \hat{a}_X \langle 1| + \lambda_X^{22}|2\rangle \hat{a}_X^\dagger \hat{a}_X \langle 2|\right)\right]\end{aligned} \tag{S4}$$

The $|1\rangle\langle 1|$ block in the electronic index will be explicitly given by:

$$\begin{aligned}\hat{\rho}_0^{11} &= \frac{1}{Z} e^{-\beta\Delta} \exp\left[-\sum_X \beta\omega_X |1\rangle \left(\hat{a}_X^\dagger \hat{a}_X + \frac{1}{2}\right)\langle 1|\right] \\ &\xrightarrow{\beta\to\infty} \frac{1}{Z} e^{-\beta\Delta} \prod_X \exp\left[-\frac{1}{2}\beta\omega_X\right] |0_X,1\rangle\langle 0_X,1| \\ &= \frac{1}{1+e^{2\beta\Delta}} \prod_X \left(1-e^{-\beta\omega_X}\right)|0_X,1\rangle\langle 0_X,1| \xrightarrow{\beta\to\infty} 0\end{aligned} \tag{S5}$$

The $|2\rangle\langle 2|$ block in the electronic index will be explicitly given by:

$$\begin{aligned}\hat{\rho}^{22} &= \frac{1}{Z} e^{\beta\Delta} \exp\left[-\sum_X \beta\omega_X |2\rangle \left(\hat{a}_X^\dagger \hat{a}_X + \frac{1}{2}\right)\langle 2|\right] \\ &\xrightarrow{\beta\to\infty} \frac{1}{1+e^{-2\beta\Delta}} \prod_X \left(1-e^{-\beta\omega_X}\right)|0_X,2\rangle\langle 0_X,2| \xrightarrow{\beta\to\infty} \prod_X |0_X,2\rangle\langle 0_X,2|\end{aligned} \tag{S6}$$

Here $|0_X,i\rangle$ is the ground vibrational state of the mode X on i-th electronic state.

Section S2. Algebraic equations of motion for the surprisal

The time-evolution of the surprisal can be derived via algebraic approach using Liouville-von-Neumann equations of motion.[2, 3] Let us consider the 2D case discussed in the main text, where we have only the coupling, ν_{10a}, and one tuning mode, ν_{6a}, in the vibrational subspace. In what follows we use the notation: $\hat{a}^\dagger\hat{a} = \hat{a}_{10a}^\dagger \hat{a}_{10a}$ and $\hat{b}^\dagger\hat{b} = \hat{a}_{6a}^\dagger \hat{a}_{6a}$ for clarity. For simplicity we also skip the quadratic coupling terms in the Hamiltonian, Eq. (S1), so the Hamiltonian is written as:

$$\begin{aligned}\hat{H} &= \left(-\Delta + \frac{1}{2}\omega_{10a} + \frac{1}{2}\omega_{6a}\right)|1\rangle\langle 1| + \left(\Delta + \frac{1}{2}\omega_{10a} + \frac{1}{2}\omega_{6a}\right)|2\rangle\langle 2| \\ &+ \omega_{10a}|1\rangle \hat{a}^\dagger \hat{a}\langle 1| + \omega_{10a}|2\rangle \hat{a}^\dagger \hat{a}\langle 2| + \omega_{6a}|1\rangle \hat{b}^\dagger \hat{b}\langle 1| + \omega_{6a}|2\rangle \hat{b}^\dagger \hat{b}\langle 2| + \\ &+ \kappa_1|1\rangle(\hat{b}+\hat{b}^\dagger)\langle 1| + \kappa_2|2\rangle(\hat{b}+\hat{b}^\dagger)\langle 2| + \kappa_{12}\left(|1\rangle(\hat{a}+\hat{a}^\dagger)\langle 2| + |2\rangle(\hat{a}+\hat{a}^\dagger)\langle 1|\right)\end{aligned} \tag{S7}$$

$$\kappa_1 = \frac{1}{\sqrt{2}}\alpha_{6a} \qquad \kappa_2 = \frac{1}{\sqrt{2}}\beta_{6a} \qquad \kappa_{12} = \frac{1}{\sqrt{2}}\gamma_{10a}$$

The propagation of the surprisal in time is determined by the equation of motion:[2]

$$i\frac{\partial \hat{I}(t)}{\partial t}=\left[\hat{H},\hat{I}(t)\right] \tag{S8}$$

In the case when the algebra is closed we can expand the time-dependent surprisal as a linear superposition of the time-independent constraints, $\hat{I}(t)=\sum_k \lambda_k(t)\hat{A}_k$. This allows us to derive the equations of motion for the Lagrange multipliers:

$$i\sum_k \frac{\partial \lambda_k(t)}{\partial t}\hat{A}_k=\sum_k \lambda_k(t)\left[\hat{H},\hat{A}_k\right]=i\sum_{k,s}\lambda_k(t)g_{ks}\hat{A}_s \tag{S9}$$

Here we assume:

$$\left[\hat{H},\hat{A}_k\right]=i\sum_s g_{ks}\hat{A}_s \tag{S10}$$

Collecting the coefficients in front of the same operators on the right and left side of Eq. (S9) we can get the equation of motion for the Lagrange multipliers:

$$\frac{\partial \lambda_m(t)}{\partial t}=\sum_k \lambda_k(t)g_{km} \tag{S11}$$

In this paper however we consider the dynamics when the algebra is not closed: there is no finite set of operators that satisfy Eq. (S10). Let us show it explicitly. Following the definition for the initial surprisal, Eq. (S2), we first consider the initial constraints:

$$\hat{A}_{\mathrm{I}}^{11}\equiv|1\rangle\langle 1|:\left[\hat{H},|1\rangle\langle 1|\right]=-\kappa_{12}\left(|1\rangle(\hat{a}+\hat{a}^{\dagger})\langle 2|-|2\rangle(\hat{a}+\hat{a}^{\dagger})\langle 1|\right) \tag{S12}$$

$$\hat{A}_{\mathrm{I}}^{22}\equiv|2\rangle\langle 2|:\left[\hat{H},|2\rangle\langle 2|\right]=\kappa_{12}\left(|1\rangle(\hat{a}+\hat{a}^{\dagger})\langle 2|-|2\rangle(\hat{a}+\hat{a}^{\dagger})\langle 1|\right) \tag{S13}$$

$$\begin{aligned}\hat{A}_{a^\dagger a}^{11}&\equiv|1\rangle\hat{a}^{\dagger}\hat{a}\langle 1|:\left[\hat{H},|1\rangle\hat{a}^{\dagger}\hat{a}\langle 1|\right]=-\kappa_{12}\left(|1\rangle\hat{a}^{\dagger}\hat{a}(\hat{a}+\hat{a}^{\dagger})\langle 2|-|2\rangle(\hat{a}+\hat{a}^{\dagger})\hat{a}^{\dagger}\hat{a}\langle 1|\right)\\&=-\kappa_{12}\left(|1\rangle(\hat{a}^{\dagger}\hat{a}^{2}+\hat{a}^{\dagger}\hat{a}\hat{a}^{\dagger})\langle 2|-|2\rangle(\hat{a}\hat{a}^{\dagger}\hat{a}+(\hat{a}^{\dagger})^{2}\hat{a})\langle 1|\right)\end{aligned} \tag{S14}$$

$$\begin{aligned}\hat{A}_{a^\dagger a}^{22}&\equiv|2\rangle\hat{a}^{\dagger}\hat{a}\langle 2|:\left[\hat{H},|2\rangle\hat{a}^{\dagger}\hat{a}\langle 2|\right]=\kappa_{12}\left(|1\rangle(\hat{a}+\hat{a}^{\dagger})\hat{a}^{\dagger}\hat{a}\langle 2|-|2\rangle\hat{a}^{\dagger}\hat{a}(\hat{a}+\hat{a}^{\dagger})\langle 1|\right)\\&=\kappa_{12}\left(|1\rangle(\hat{a}\hat{a}^{\dagger}\hat{a}+(\hat{a}^{\dagger})^{2}\hat{a})\langle 2|-|2\rangle(\hat{a}^{\dagger}\hat{a}^{2}+\hat{a}^{\dagger}\hat{a}\hat{a}^{\dagger})\langle 1|\right)\end{aligned} \tag{S15}$$

$$\hat{A}_{b^\dagger b}^{11}\equiv|1\rangle\hat{b}^{\dagger}\hat{b}\langle 1|:\left[\hat{H},|1\rangle\hat{b}^{\dagger}\hat{b}\langle 1|\right]=\kappa_1|1\rangle\left[(\hat{b}+\hat{b}^{\dagger}),\hat{b}^{\dagger}\hat{b}\right]\langle 1|=\kappa_1|1\rangle(\hat{b}-\hat{b}^{\dagger})\langle 1| \tag{S16}$$

$$\hat{A}_{b^\dagger b}^{22}\equiv|2\rangle\hat{b}^{\dagger}\hat{b}\langle 2|:\left[\hat{H},|2\rangle\hat{b}^{\dagger}\hat{b}\langle 2|\right]=\kappa_2|2\rangle\left[(\hat{b}+\hat{b}^{\dagger}),\hat{b}^{\dagger}\hat{b}\right]\langle 2|=\kappa_2|2\rangle(\hat{b}-\hat{b}^{\dagger})\langle 2| \tag{S17}$$

We see that we have to expand our initial set of 6 constraints with 16 additional operators: $\{\hat{a},\hat{a}^{\dagger},\hat{a}^{\dagger}\hat{a}^{2},\hat{a}^{\dagger}\hat{a}\hat{a}^{\dagger},\hat{a}\hat{a}^{\dagger}\hat{a},(\hat{a}^{\dagger})^{2}\hat{a}\}\otimes\{|1\rangle\langle 2|,|2\rangle\langle 1|\}$ and $\{\hat{b},\hat{b}^{\dagger}\}\otimes\{|1\rangle\langle 1|,|2\rangle\langle 2|\}$. Already for the

simple 2D case we need more than 20 operators to describe the first order approximation. It therefore makes sense to determine a set of dominant constraints. The not small price is that unlike what is happening here we only obtain a matrix representation of the constraints and it is not always easy to translate a sparse matrix to an observable with an intuitive physical meaning. Towards the end of section S3 there are many concrete examples of this aspect, for example, Figs. S15-S19.

The failure of the algebra to close remains also in higher orders. Considering equations of motion for this bigger set we will need to further expand the set. For example:

$$\begin{aligned}\hat{A}_a^{12} \equiv |1\rangle \hat{a} \langle 2| : \left[\hat{H}, |1\rangle \hat{a} \langle 2|\right] = -2\Delta |1\rangle \hat{a} \langle 2| &+ \omega_{10a}\left(|1\rangle \hat{a}^\dagger \hat{a}^2 \langle 2| - |1\rangle \hat{a}\hat{a}^\dagger \hat{a} \langle 2|\right) \\ &+ (\kappa_1 - \kappa_2)|1\rangle (\hat{b} + \hat{b}^\dagger)\hat{a}\langle 2| \\ &+ \kappa_{12}\left(|2\rangle (\hat{a}^2 + \hat{a}^\dagger \hat{a})\langle 2| - |1\rangle (\hat{a}^2 + \hat{a}\hat{a}^\dagger)\langle 1|\right)\end{aligned} \quad \text{(S18)}$$

gives additional 4 operators $\left\{\hat{b}\hat{a}, \hat{b}^\dagger \hat{a}\right\} \otimes |1\rangle\langle 2|$ and $\hat{a}^2 \otimes \{|1\rangle\langle 1|, |2\rangle\langle 2|\}$.

Section S3. Additional figures

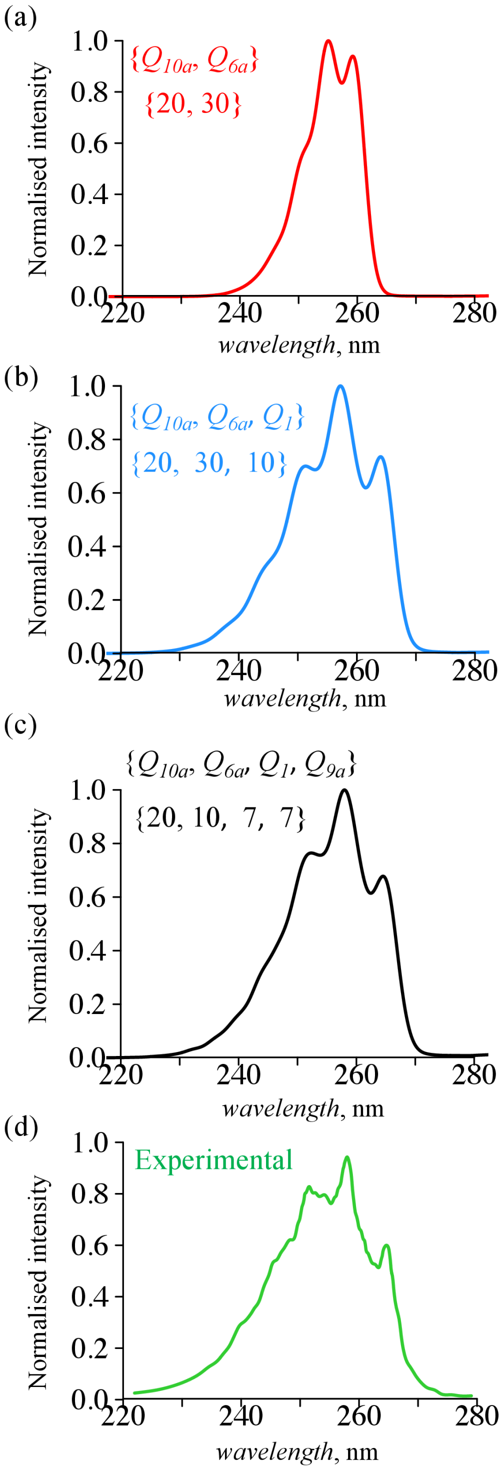

Figure S1. Comparison of the computed absorption spectra for model Hamiltonians in 2D (a), 3D (b), and 4D (c) bases with the experimental spectrum from Ref. 4 (d). The number of the vibrational basis functions taken for the normal mode representation is shown on each panel. The spectral envelope has been obtained by convoluting the stick spectrum with a Gaussian of FWHM = 71 meV.

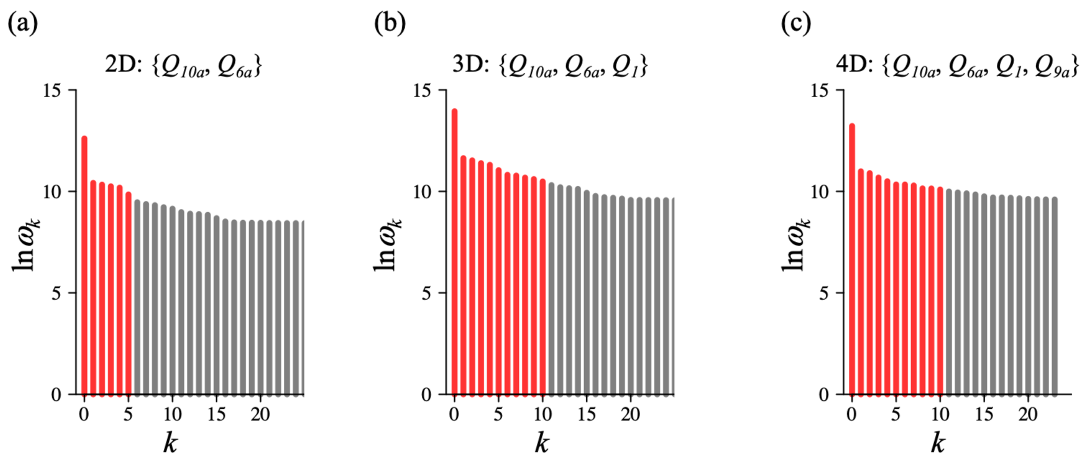

Figure S2. Singular eigenvalues in the SVD expansion of the time-dependent surprisal, Eq. (7) of the main text, for 2D (a), 3D (b) and 4D (c) cases of the nuclear degrees of freedom shown on a logarithmic scale. Normal mode coordinates involved in the dynamics are given on each panel. Red lines highlight the eigenvalues of the dominant constraints that we include in the approximation of the surprisal. In all three cases the first eigenvalue ω_0 is several orders of magnitude larger compare to other ω_k.

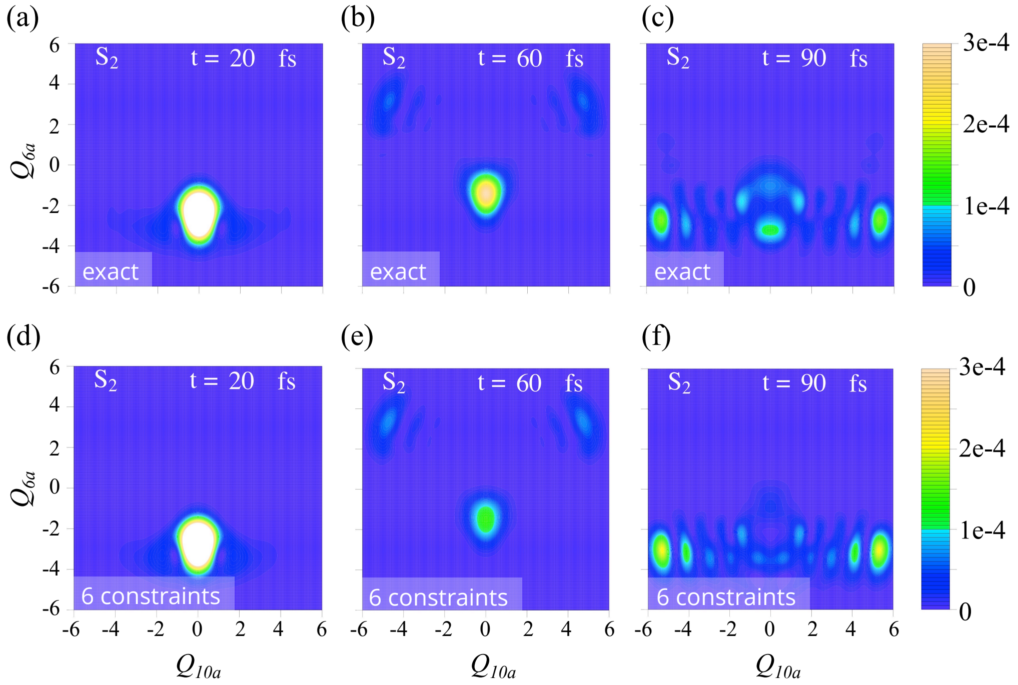

Figure S3. Snapshots of the population distribution in the 2D case of the dynamics on the bright, $S_2(\pi\pi^*)$, electronic state as a function of two nuclear coordinates: coupling mode, Q_{10a}, and tuning mode, Q_{6a} taken at different times of the dynamics, shown in the top right corner on each panel. Exact results (a-c) are compared with the approximate computation (d-f) via 6 dominant constraints.

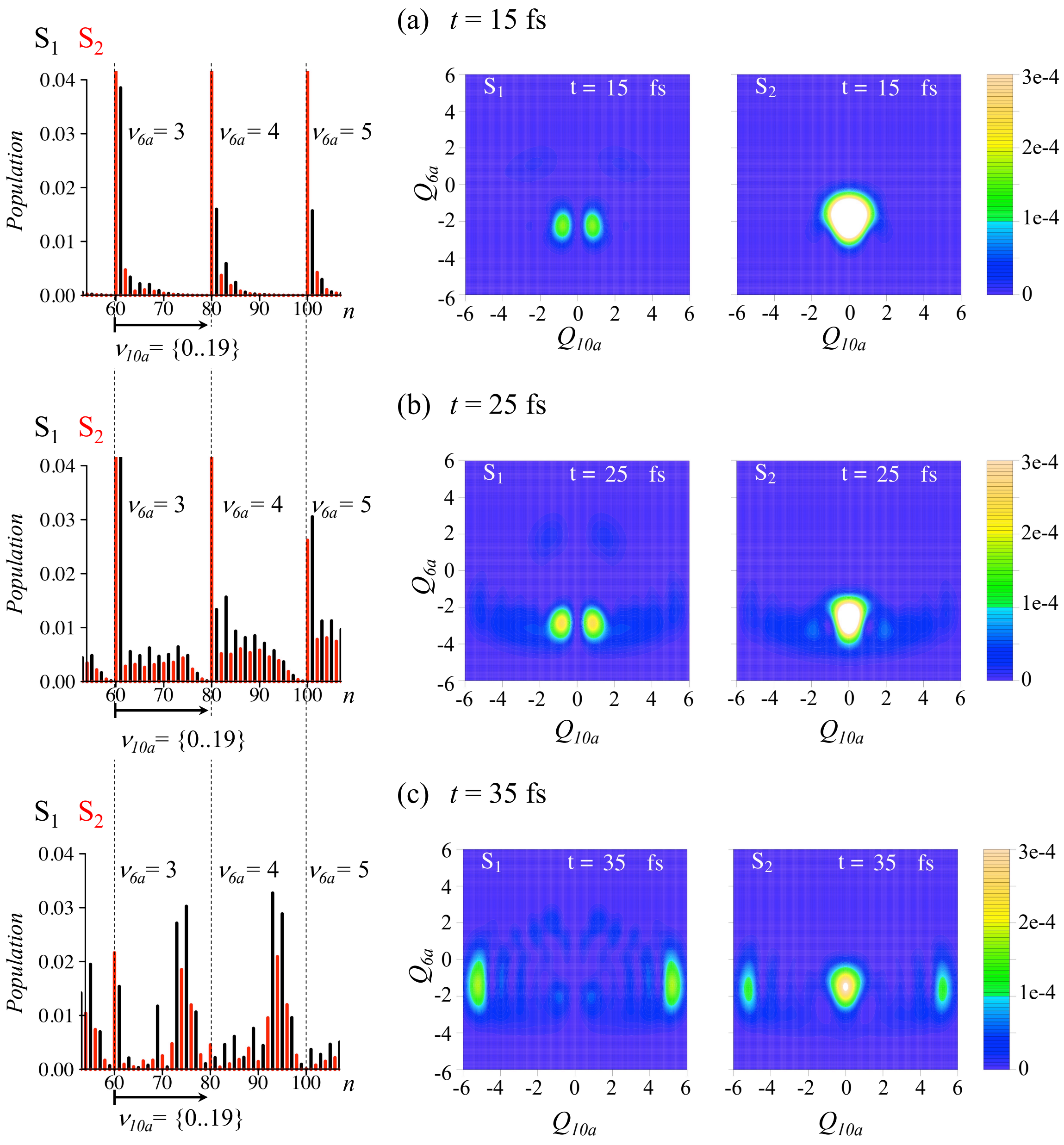

Figure S4. Snapshots of the early time dynamics at times when a nodal structure of the nuclear wave packets appears in both electronic states. The snapshots are taken at 15fs (a), 25fs (b) and 35fs(c) of the dynamics. Shown are the results of the exact 2D-surprisal propagation. Left panels provide population in the vibrational state basis for S_1 (black lines) and S_2 (red lines) electronic states. The index of the $\{Q_{10a}, Q_{6a}\}$ vibrational states is defined as $n = 20 \cdot v_{6a} + v_{10a} + 1$, where v_{6a} is the vibrational quantum number of the tuning mode, and v_{10a} is the quantum number of the coupling mode. Plotted is the population for the $v_{6a} = 3,4,5$ and $v_{10a} = 0,...,19$. Right panels present S_1 and S_2 population distribution as a function of two nuclear coordinates for the same time-step of the dynamics: coupling mode, Q_{10a}, and tuning mode, Q_{6a}.

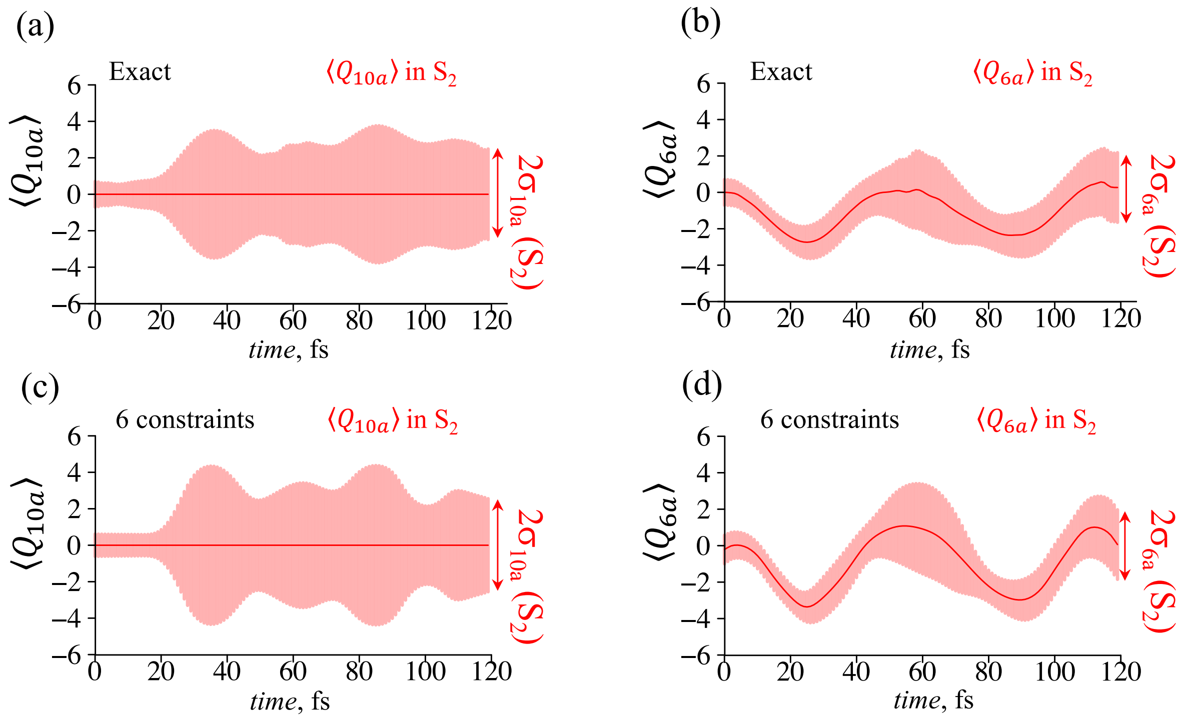

Figure S5. Dynamics of the mean values $\langle Q_{10a} \rangle$ (a, c) and $\langle Q_{6a} \rangle$ (b, d) (solid lines) and their respective dispersion σ_{10a} and σ_{6a} (red area covering $\langle Q_X \rangle \pm \sigma_X$) computed in the S_2 electronic state for the 2D-case. Exact computations (a, b) are compared to the approximation (c, d) given by 6 dominant constraints.

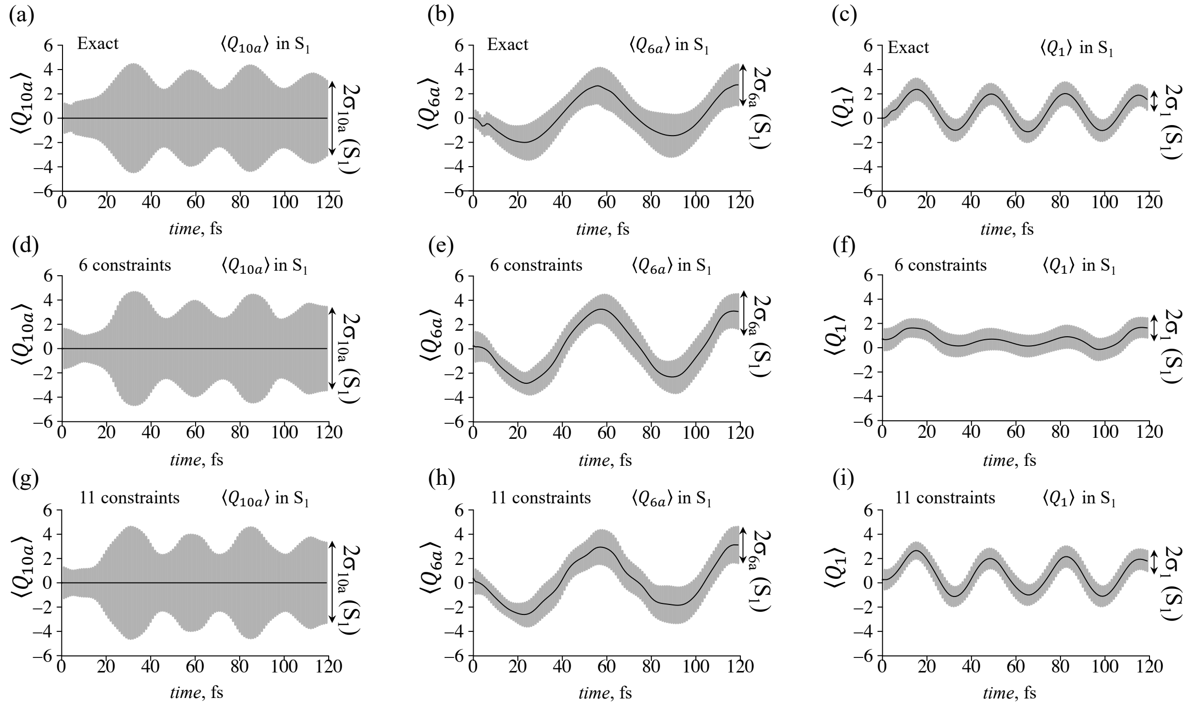

Figure S6. Dynamics of the mean values $\langle Q_{10a} \rangle$ (a, d, g), $\langle Q_{6a} \rangle$ (b, e, h) and $\langle Q_1 \rangle$ (c, f, i) (solid lines) and their respective dispersion σ_{10a}, σ_{6a} and σ_1 (gray area covering $\langle Q_X \rangle \pm \sigma_X$) computed in the S_1 electronic state for the 3D-case. Exact computations (a-c) are compared to the approximations given by 6 (d-f) and 11 (g-i) dominant constraints.

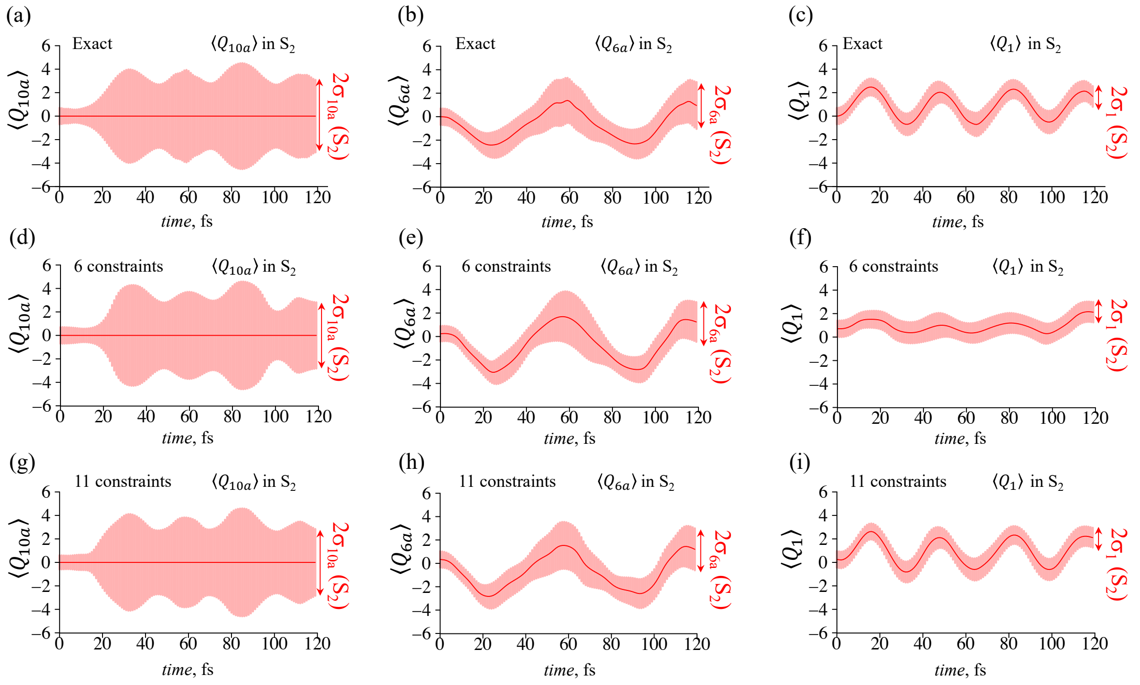

Figure S7. Dynamics of the mean values $\langle Q_{10a} \rangle$ (a, d, g), $\langle Q_{6a} \rangle$ (b, e, h) and $\langle Q_1 \rangle$ (c, f, i) (solid lines) and their respective dispersion σ_{10a}, σ_{6a} and σ_1 (red area covering $\langle Q_X \rangle \pm \sigma_X$

) computed in the S_2 electronic state for the 3D-case. Exact computations (a-c) are compared to the approximations given by 6 (d-f) and 11 (g-i) dominant constraints.

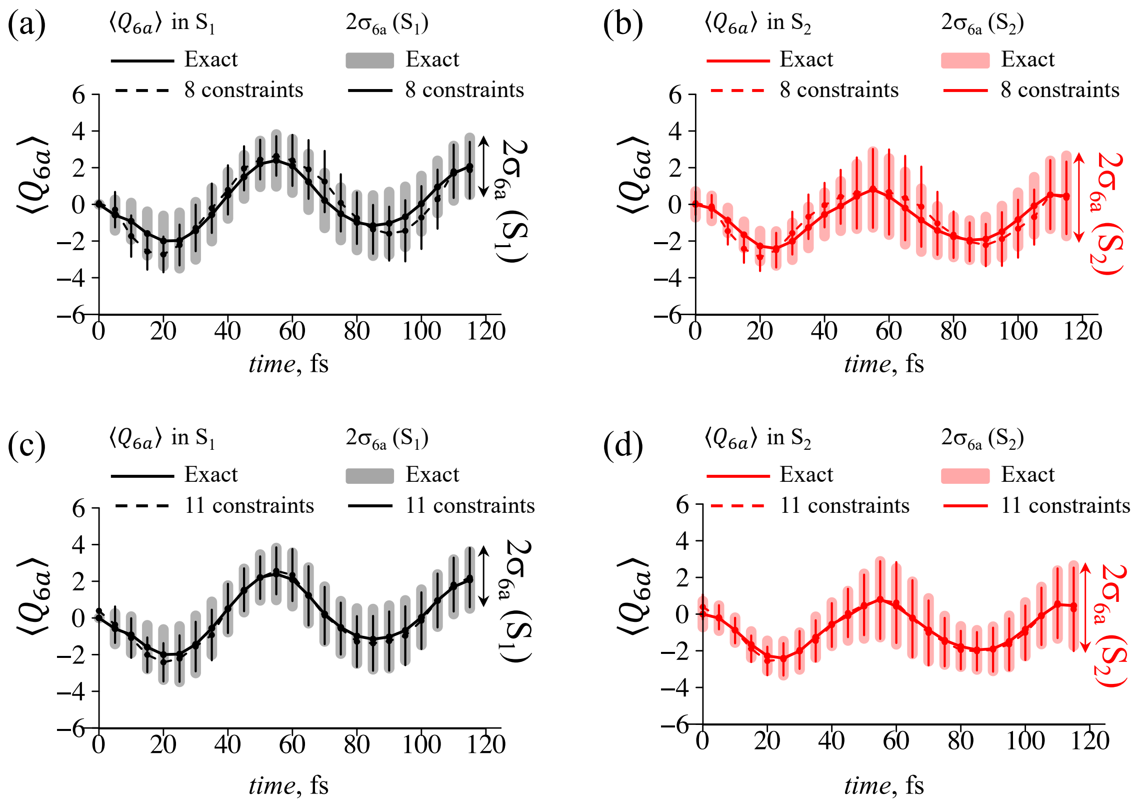

FIG S8. Mean and dispersion of the tuning normal mode, $\langle Q_{6a} \rangle \pm \sigma_{6a}$, in S_1 (a, c) and S_2 (b, d) electronic states in 4D-dynamics. Approximate results provided by 8 (a, b) and 11 (c, d) dominant constraints are compared to the results of the exact computation. Approximate mean values – dashed lines, exact mean values – solid lines; approximate dispersion - black sticks, exact dispersion – colored area.

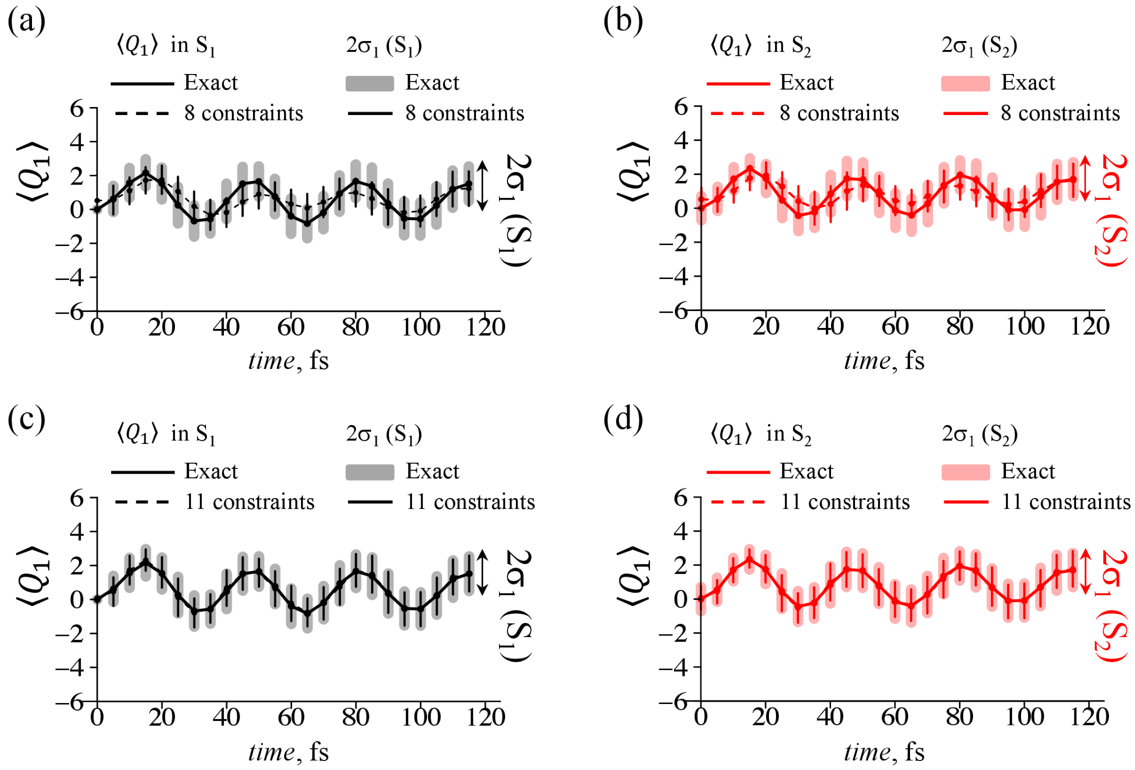

Figure S9. Mean and dispersion of the tuning normal mode, $\langle Q_1 \rangle \pm \sigma_1$, in S_1 (a, c) and S_2 (b, d) electronic states in 4D-dynamics. Approximate results provided by 8 (a, b) and 11 (c, d) dominant constraints are compared to the results of the exact computation. Approximate mean values – dashed lines, exact mean values – solid lines; approximate dispersion – black sticks, exact dispersion – colored area.

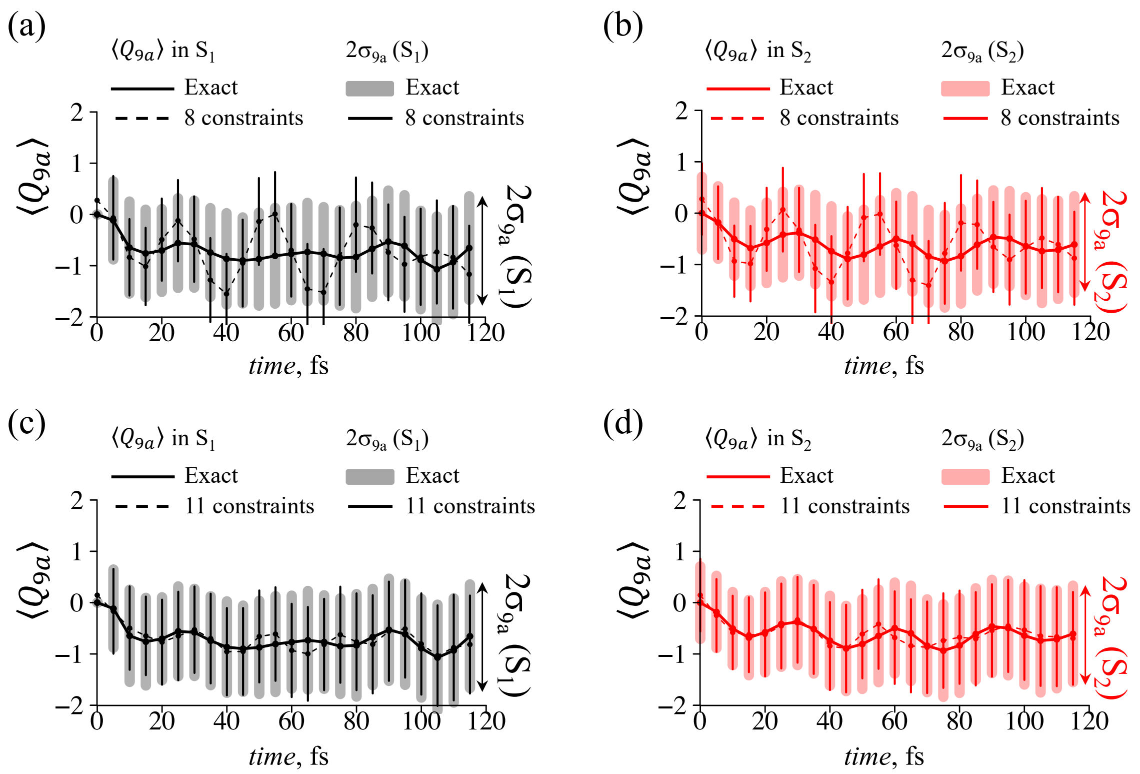

Figure S10. Mean and dispersion of the tuning normal mode, $\langle Q_{9a} \rangle \pm \sigma_{9a}$, in S_1 (a, c) and S_2 (b, d) electronic states in 4D-dynamics. Approximate results provided by 8 (a, b) and 11 (c, d) dominant constraints are compared to the results of the exact computation. Approximate mean values – dashed lines, exact mean values – solid lines; approximate dispersion – black sticks, exact dispersion – colored area.

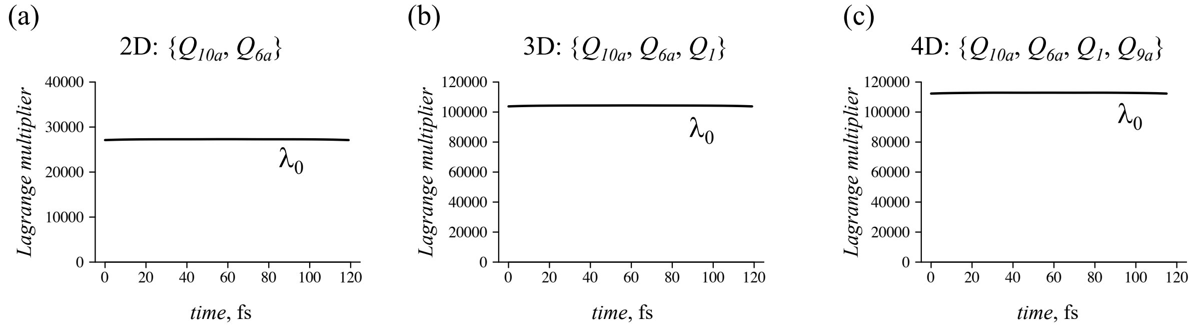

Figure S11. Lagrange multiplier for the leading term $\mathbf{G_0}$, $\lambda_0(t)$, in the case of 2D (a), 3D (b), and 4D (c) nuclear dynamics. The Lagrange multiplier is constant in time up to 1% of its magnitude.

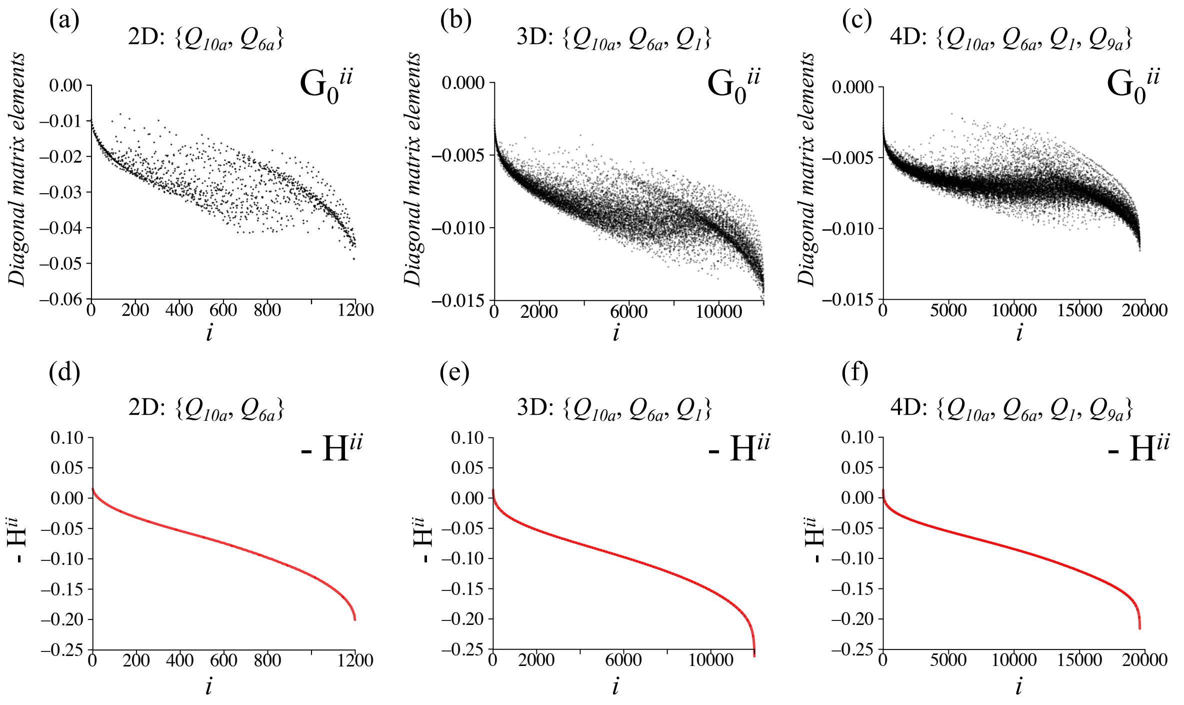

Figure S12. Diagonal matrix elements of $\mathbf{G}_0$ (a – c) and of the Hamiltonian (d-f) in the basis of the Hamiltonian eigenstates as a function of the eigenstate index *i* for 2D (a, d), 3D (b,e) and 4D(c,f) dynamics.

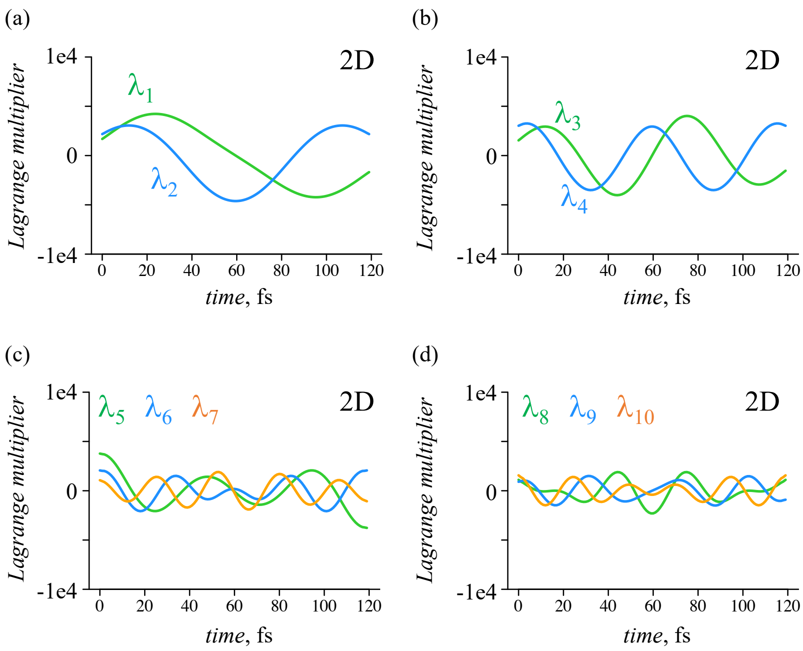

Figure S13. Time-dependent Lagrange multipliers, $\lambda_k(t)$, in the case of 2D nuclear dynamics in two electronic states for an extended set of 11 dominant constraints: (a) $\lambda_1(t)$ and $\lambda_2(t)$; (b) $\lambda_3(t)$ and $\lambda_4(t)$; (c) $\lambda_5(t), \lambda_6(t)$ and $\lambda_7(t)$; (d) $\lambda_8(t), \lambda_9(t)$ and $\lambda_{10}(t)$. 2D-vibrational space involves $\{Q_{10a}, Q_{6a}\}$ normal modes.

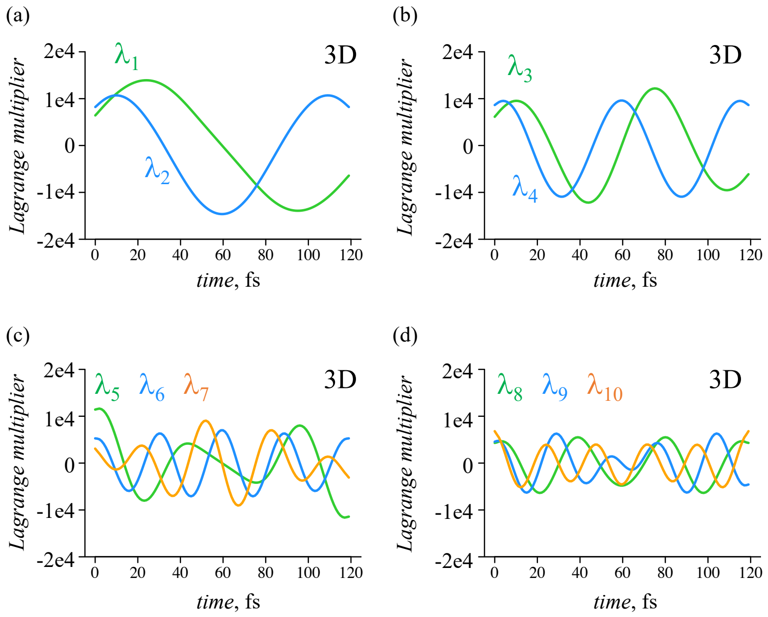

Figure S14. Time-dependent Lagrange multipliers, $\lambda_k(t)$, in the case of 3D nuclear dynamics in two electronic states for an extended set of 11 dominant constraints: (a) $\lambda_1(t)$ and $\lambda_2(t)$; (b) $\lambda_3(t)$ and $\lambda_4(t)$; (c) $\lambda_5(t), \lambda_6(t)$ and $\lambda_7(t)$; (d) $\lambda_8(t), \lambda_9(t)$ and $\lambda_{10}(t)$. 3D-vibrational space involves $\{Q_{10a}, Q_{6a}, Q_1\}$ normal modes.

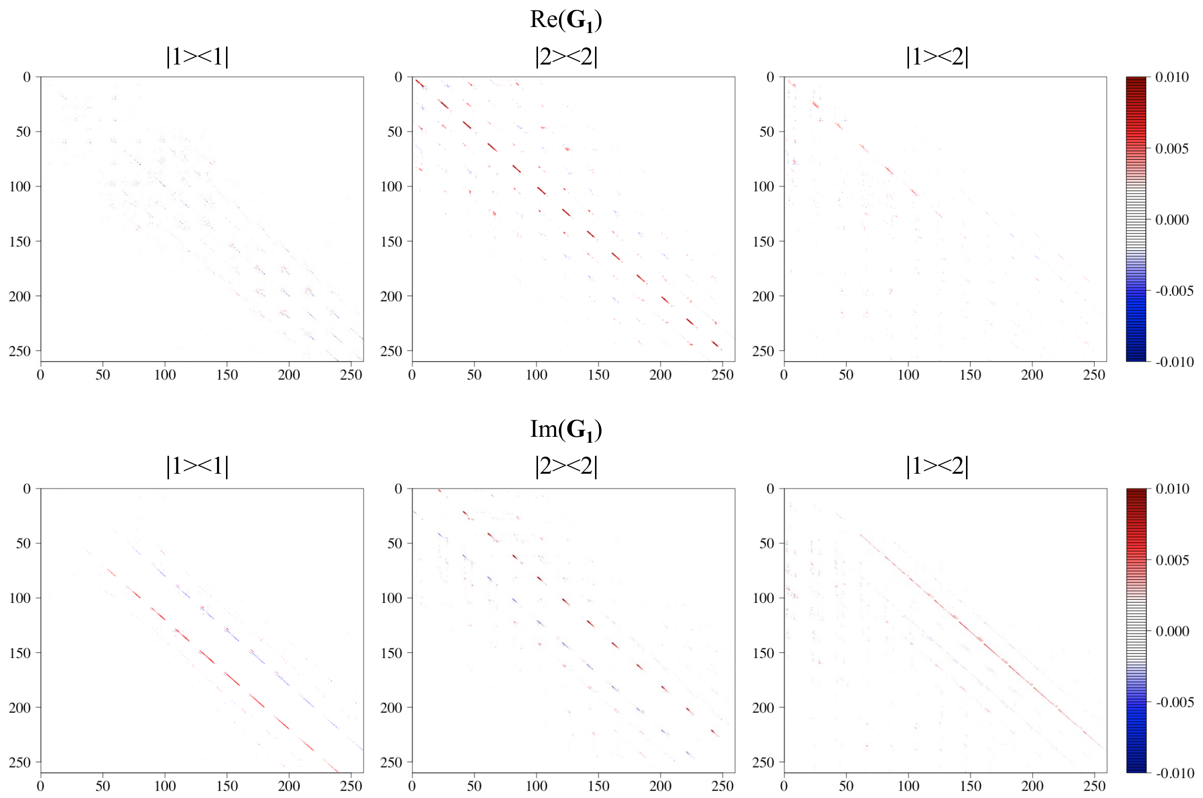

Figure S15. Real (top row) and imaginary part (bottom row) of the matrix elements in the $|1\rangle\langle 1|, |2\rangle\langle 2|$ and $|1\rangle\langle 2|$ blocks (in the electronic index) for the $\mathbf{G}_1$ constraint. Corresponding indices of the matrix elements, $20 \cdot v_{6a} + v_{10a} + 1$, spanning the range of the vibrational states involved in the dynamics are given as axes. Matrix elements with 1-260 indices correspond to the vibrational basis function with $v_{6a} = 0,\ldots,12$ of the tuning mode and $v_{10a} = 0,\ldots,19$ of the coupling mode.

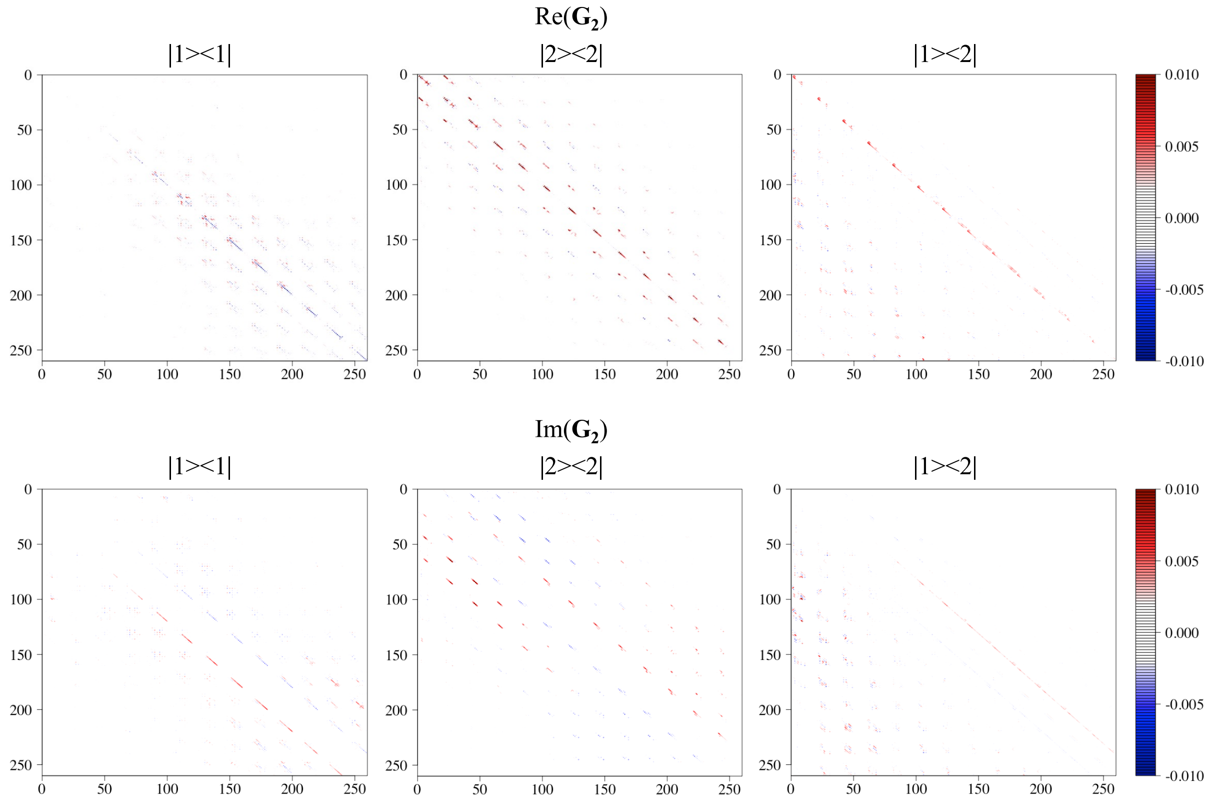

Figure S16. As in Fig. S15 but for the $\mathbf{G}_2$ constraint. Corresponding indices of the matrix elements, $20 \cdot v_{6a} + v_{10a} + 1$, spanning the range of the vibrational states involved in the dynamics are given as axes. Matrix elements with 1-260 indices correspond to the vibrational basis function with $v_{6a} = 0,...,12$ of the tuning mode and $v_{10a} = 0,...,19$ of the coupling mode.

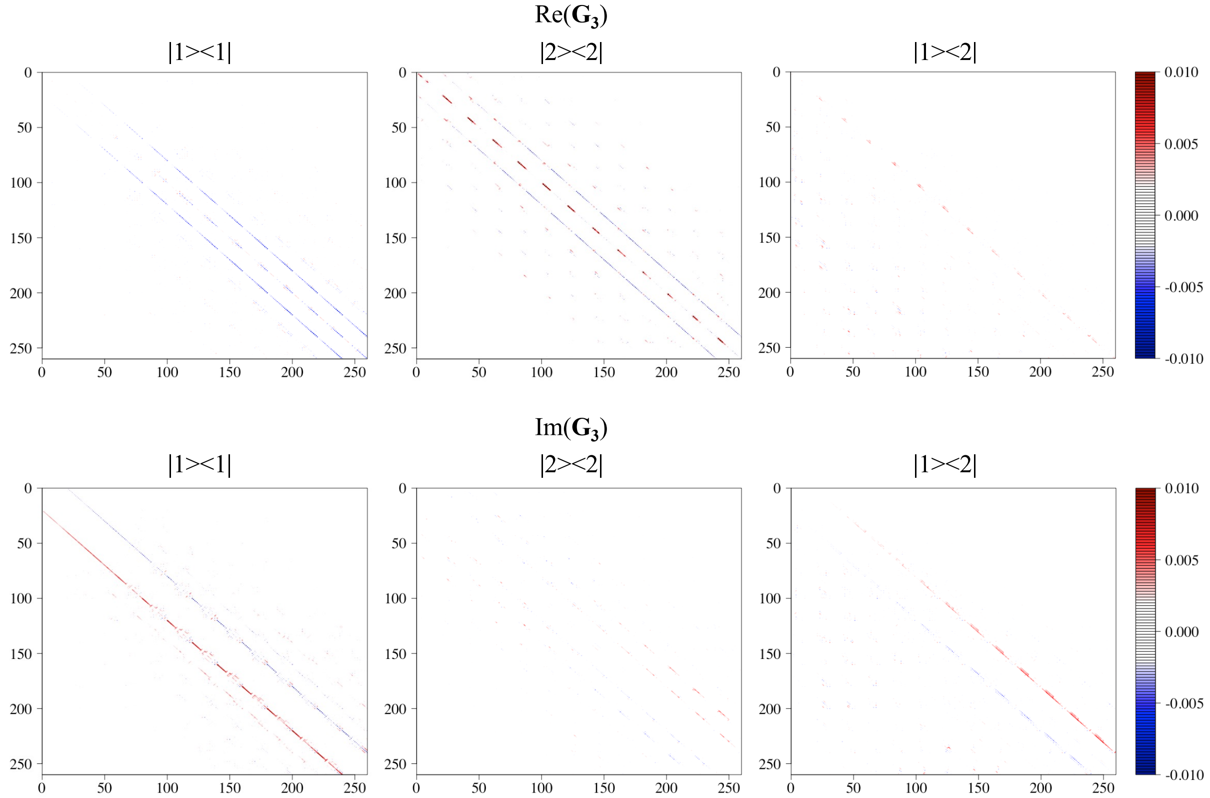

Figure S17. Same as Figs. S15 and S16 for the $\mathbf{G}_3$ constraint. Corresponding indices of the matrix elements, $20 \cdot v_{6a} + v_{10a} + 1$, spanning the range of the vibrational states involved in the dynamics are given as axes. Matrix elements with 1-260 indices correspond to the vibrational basis function with $v_{6a} = 0,...,12$ of the tuning mode and $v_{10a} = 0,...,19$ of the coupling mode.

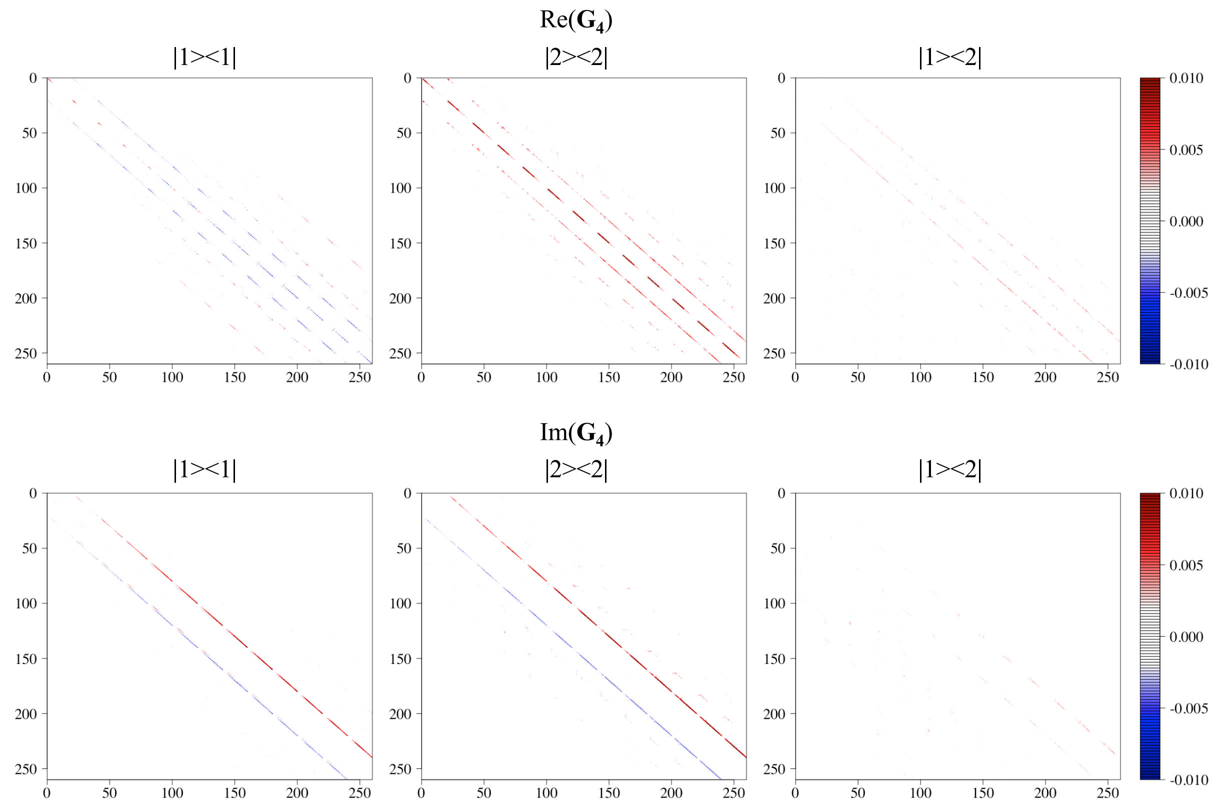

Figure S18. Same as Figs. S15-S17 for the **G**4 constraint. Corresponding indices of the matrix elements, $20 \cdot v_{6a} + v_{10a} + 1$, spanning the range of the vibrational states involved in the dynamics are given as axes. Matrix elements with 1-260 indices correspond to the vibrational basis function with $v_{6a} = 0,...,12$ of the tuning mode and $v_{10a} = 0,...,19$ of the coupling mode.

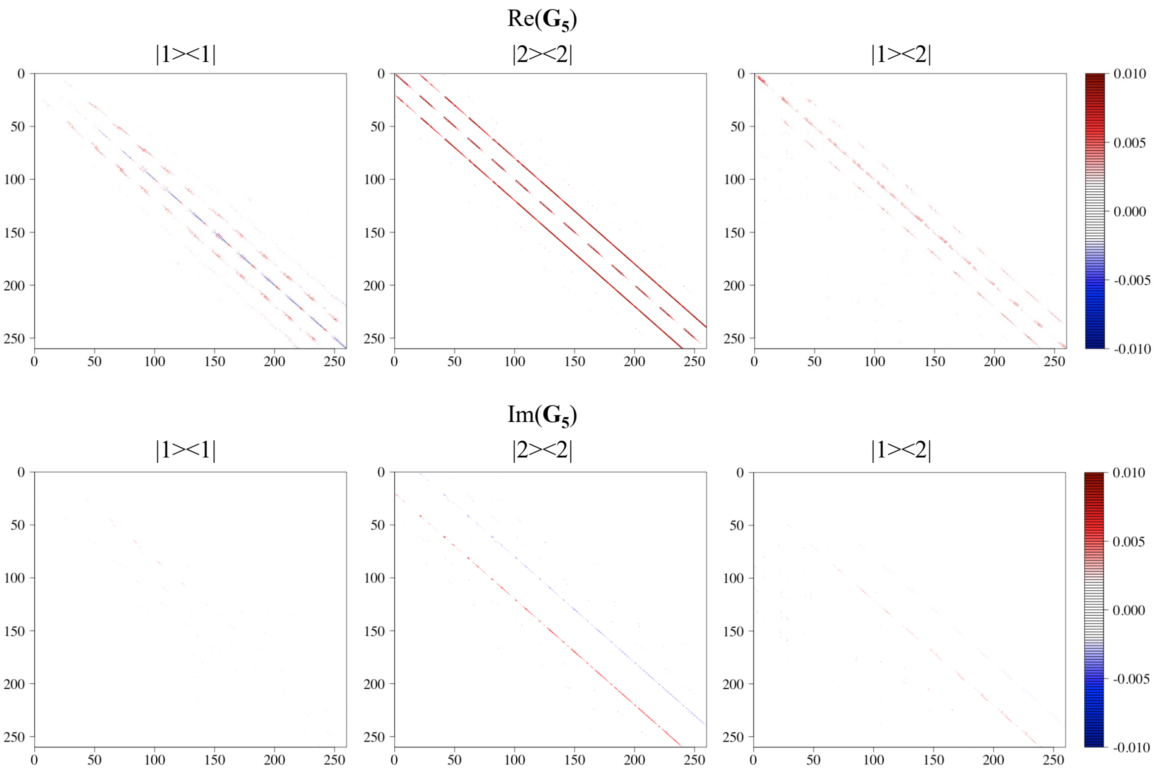

Figure S19. Same as Figs. S15-S18 for the **G**5 constraint. Corresponding indices of the matrix elements, $20\cdot v_{6a}+v_{10a}+1$, spanning the range of the vibrational states involved in the dynamics are given as axes. Matrix elements with 1-260 indices correspond to the vibrational basis function with $v_{6a}=0,\ldots,12$ of the tuning mode and $v_{10a}=0,\ldots,19$ of the coupling mode.

Movie S1. The dynamics of the population distribution on S_1 electronic state as a function of two nuclear coordinates along the 120fs time-range for 2D case. Left panel shows results of the 6 constraint SVD based approximation, while the right panel presents exact dynamics. Normal mode coordinates are: q1 – coupling mode Q_{10a}, q2 – tuning mode Q_{6a}.

Movie S2. The dynamics of the population distribution on S_2 electronic state as a function of two nuclear coordinates along the 120fs time-range for 2D case. Left panel shows results of the 6 constraint SVD based approximation, while the right panel presents exact dynamics. Normal mode coordinates are: q1 – coupling mode Q_{10a}, q2 – tuning mode Q_{6a}.

Movie S3. Population dynamics on both electronic states for different vibrational basis functions in the 2D case: S_1 population - black sticks, S_2 population red sticks. The index of the $\{Q_{10a},Q_{6a}\}$ vibrational states is defined as $n=20\cdot v_{6a}+v_{10a}+1$, where v_{6a} is the

vibrational quantum number of the tuning mode, and v_{10a} is the quantum number of the coupling mode. Plotted is the population for the v_{6a}=0,…,14 and $v_{10a}=0,...,19$.

Section S4. Dominant constraints identified for the long time-span of the dynamics

In this section we provide a brief discussion for the 2D case of the dynamics extended in time up to 1ps, giving in total L=1000 time-points in the matrix of the time-dependent surprisal, Figure 1(a). The set of singular eigenvalues calculated for this case in the SVD procedure is shown in Figure S20. Although the number of the dominant constraints increased to about 40 terms (compare to 6 dominant constraints in the 120fs dynamics), the order of magnitude gap between the first eigenvalue, ω_0, and the rest set of eigenvalues is still present. The few first constraints, $\mathbf{G}_1$-$\mathbf{G}_4$, are shown in Figs. S21-S22.

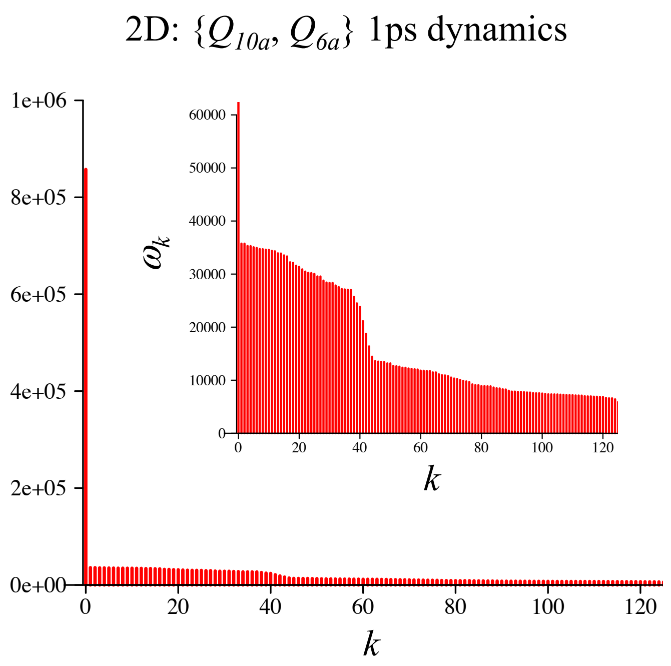

Figure S20. Singular eigenvalues in the SVD expansion of the time-dependent surprisal, Eq. (7) of the main text, for 2D case dynamics extended up to 1ps, the number of the time-points considered, L = 1000. The number of dominant constraints grows up to 40, which is still acceptable, compare to the exact description by 1000 terms.

In our previous study[5] we also analyzed the dominant constraints representation for the long-time range limit for 1D-anharmonic systems. We found that it converges to the finite basis representation, where each constraint is defined as $|m\rangle\langle n|$ Gelfand operator for a single

pair of eigenstates *m* and *n*. The corresponding Lagrange multipliers match accordingly the values of the matrix elements of the exact surprisal $I_{mn}(t)$ in the eigenstate basis. Our understanding is that for the larger time ranges, larger number of time points in the SVD, energy spacings of the coherences are all resolved. This prevents grouping of different eigenstates under the same time-dependent Lagrange multiplier. Similar picture we see in the 2D dynamics in pyrazine. Almost degenerate singular eigenvalues ω_k for $k = 1,\ldots,40$ correspond to the constraints that have more localized contributions from only few eigenstates, this is seen in particular for large *k,* Figure S22.

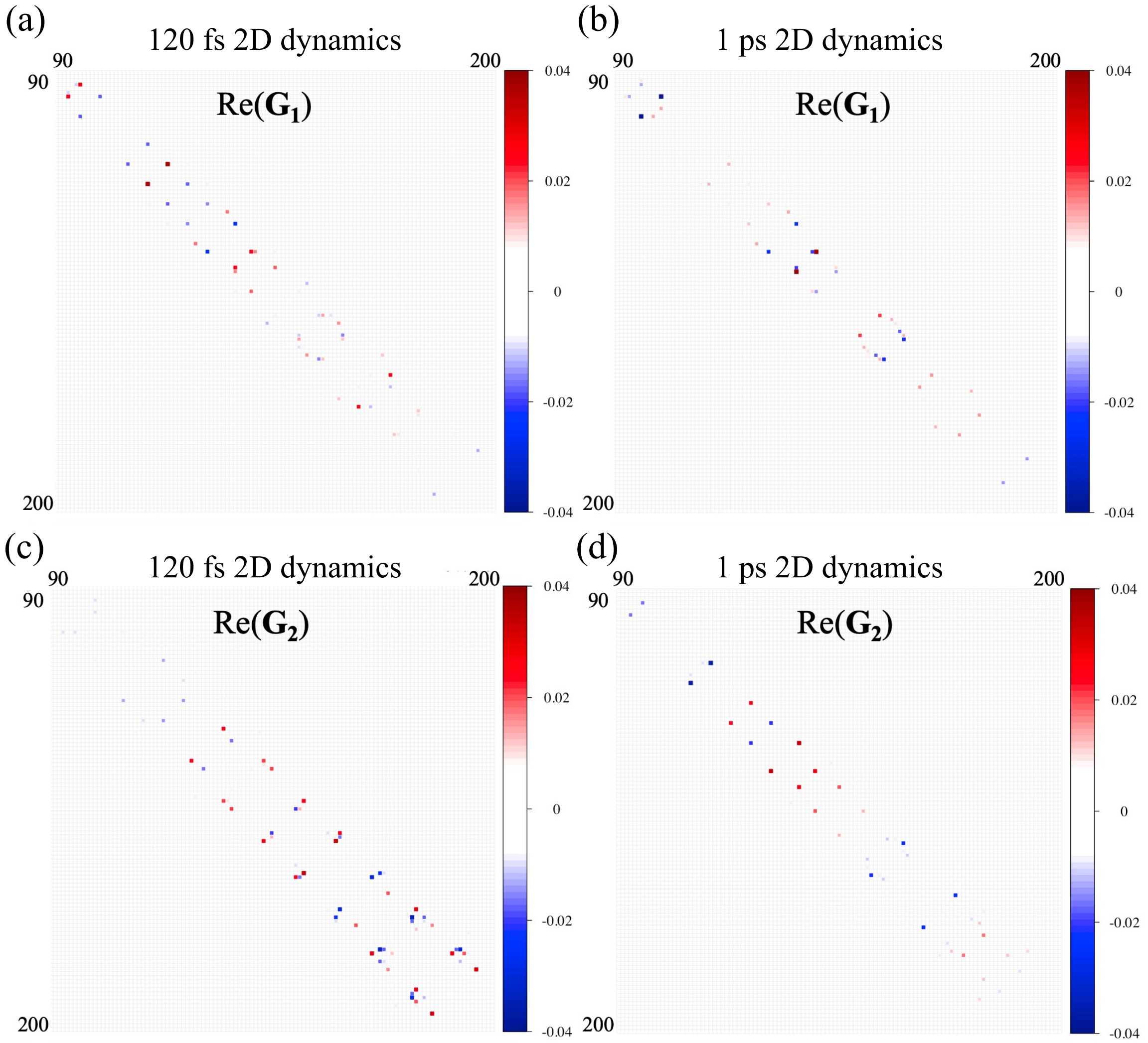

Figure S21. Comparison of the $\mathbf{G}_1$ and $\mathbf{G}_2$ constraints in the eigenstate basis representation calculated for the 2D-case of the dynamics during 120 fs (a, c) and 1ps (b, d). Shown is the real part of the matrix elements for the range of largely populated eigenstates.

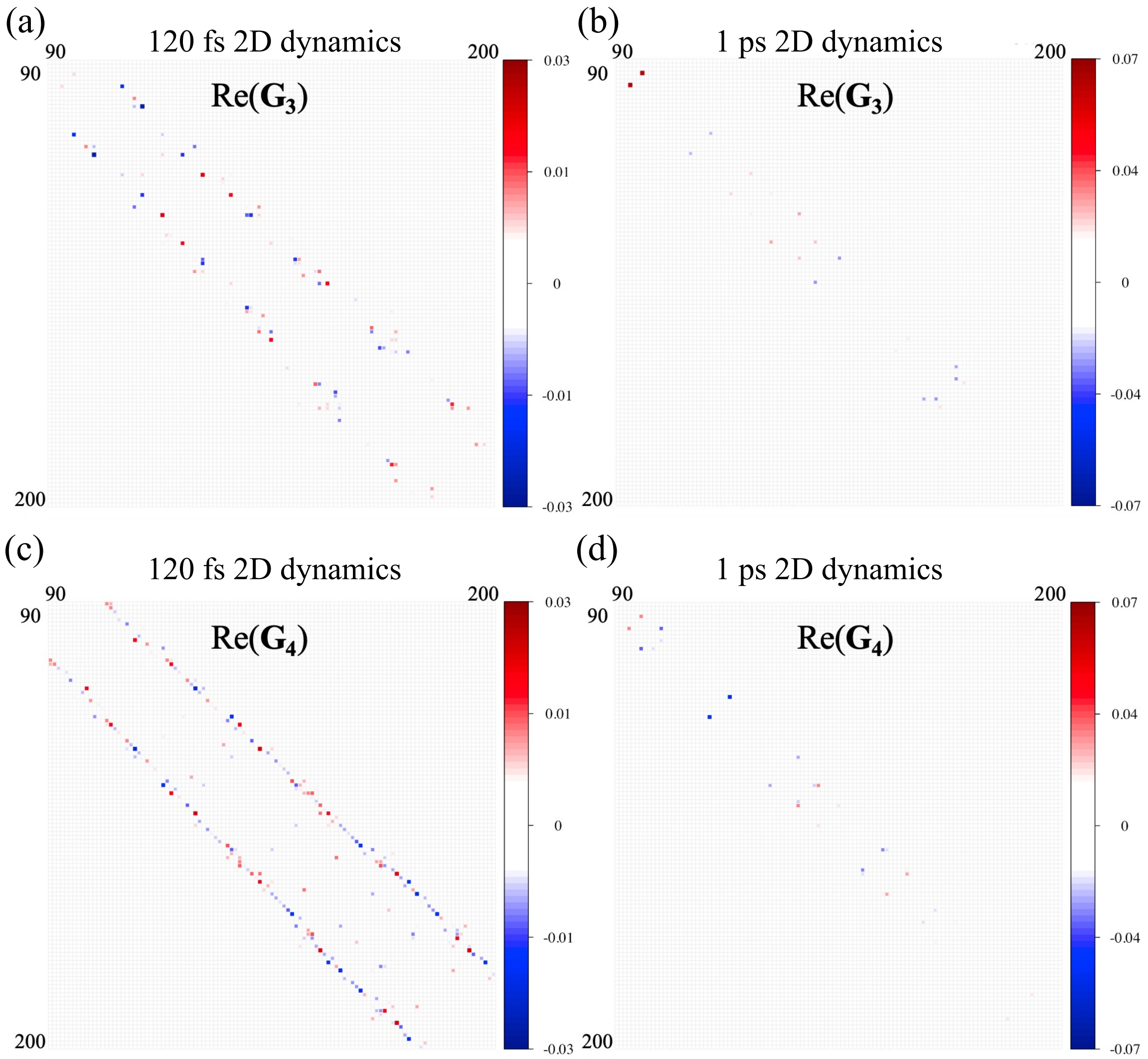

Figure S22. Comparison of the $\mathbf{G}_3$ and $\mathbf{G}_4$ constraints in the eigenstate basis representation calculated for the 2D-case of the dynamics during 120 fs (a, c) and 1ps (b, d). Shown is the real part of the matrix elements for the range of largely populated eigenstates.

References

[1]A. Raab, G. A. Worth, H.-D. Meyer and L. S. Cederbaum, "Molecular dynamics of pyrazine after excitation to the S2 electronic state using a realistic 24-mode model Hamiltonian," J. Chem. Phys. **110**, 936-946 (1999).

[2]Y. Alhassid and R. D. Levine, "Entropy and chemical change. III. The maximal entropy (subject to constraints) procedure as a dynamical theory," J. Chem. Phys. **67**, 4321-4339 (1977).

[3]K. Komarova, F. Remacle and R. D. Levine, "Surprisal of a quantum state: Dynamics, compact representation, and coherence effects," J. Chem. Phys. **153**, 214105 (2020).

[4]I. Yamazaki, T. Murao, T. Yamanaka and K. Yoshihara, "Intramolecular electronic relaxation and photoisomerization processes in the isolated azabenzene molecules pyridine, pyrazine and pyrimidine," Faraday Discuss. Chem. Soc. **75**, 395-405 (1983).

[5]K. Komarova, F. Remacle and R. D. Levine, "Compacting the density matrix in quantum dynamics: Singular value decomposition of the surprisal and the dominant constraints for anharmonic systems," J. Chem. Phys. **155**, 204110 (2021).
